# Identifying Gene–Environment Interactions With Robust Marginal Bayesian Variable Selection

**DOI:** 10.3389/fgene.2021.667074

**Published:** 2021-12-08

**Authors:** Xi Lu, Kun Fan, Jie Ren, Cen Wu

**Affiliations:** ^1^Department of Statistics, Kansas State University, Manhattan, KS, United States; ^2^Department of Biostatistics, Indiana University School of Medicine, Indianapolis, IN, United States

**Keywords:** gene-environment interaction, marginal analysis, robust Bayesian variable selection, spike-and-slab priors, markov chain monte carlo method

## Abstract

In high-throughput genetics studies, an important aim is to identify gene–environment interactions associated with the clinical outcomes. Recently, multiple marginal penalization methods have been developed and shown to be effective in G×E studies. However, within the Bayesian framework, marginal variable selection has not received much attention. In this study, we propose a novel marginal Bayesian variable selection method for G×E studies. In particular, our marginal Bayesian method is robust to data contamination and outliers in the outcome variables. With the incorporation of spike-and-slab priors, we have implemented the Gibbs sampler based on Markov Chain Monte Carlo (MCMC). The proposed method outperforms a number of alternatives in extensive simulation studies. The utility of the marginal robust Bayesian variable selection method has been further demonstrated in the case studies using data from the Nurse Health Study (NHS). Some of the identified main and interaction effects from the real data analysis have important biological implications.

## 1. Introduction

The risk and progression of complex diseases including cancer, asthma and type 2 diabetes are associated with the coordinated functioning of genetic factors, the environmental (and clinical) factors, as well as their interactions (Hunter, 2005; Von Mutius, 2009; Cornelis and Hu, 2012; Simonds et al., 2016). The identification of important gene–environment(G×E) interactions leads to novel insight in dissecting the genetic basis of complex diseases in addition to the main effects of genetic and environmental factors. In the last two decades, searching for the important G×E interactions has been extensively conducted based on genetic association studies (Cordell and Clayton, 2005; Wu et al., 2012). One representative example is the genome-wide association study (GWAS), where the statistical significance of interaction between the environmental exposure and the genetic variant has been marginally assessed one at a time across the whole genome. Important findings are evidenced by genome-wide significant *p*-values after adjusting for multiple comparisons.

Recently, substantial efforts have been devoted to novel penalized variable selection methods for G×E studies (Zhou et al., 2021). In particular, marginal penalization has achieved very competitive performances with the aforementioned significance-based G×E analysis (Shi et al., 2014; Chai et al., 2017; Zhang et al., 2020). For example, within the framework of maximum rank correlation, Shi et al. (2014) has developed a penalization method robust to outliers and model misspecification in determining important G×E interactions one at a time. Zhang et al. (2020) has imposed hierarchical structure between the main effects and interactions in marginal identification of G×E interactions using regularization. Despite success, these studies have limitations. First, as a common tuning parameter is demanded for all the marginal models, its selection requires pooling all genes together to conduct a joint model-based cross-validation. While such a strategy is not rare, it seems not in favor of the marginal nature of the proposed G×E studies. Second, a rigorous measure to quantify uncertainty is not available. Zhang et al. (2020) has constructed 95% confidence intervals based on the observed occurrence index (OOI) values (Huang and Ma, 2010); nevertheless, this measure has been used to demonstrate stability of identified effects rather than quantifying uncertainty of penalized estimates.

These limitations have motivated us to consider Bayesian analyses. In literature, Bayesian variable selection methods have been developed for G×E analysis in multiple studies (Zhou et al., 2021). For example, with indicator model selection, Liu et al. (2015) has imposed hierarchical Bayesian variable selection for linear G×E interactions. Li et al. (2015) has proposed a Bayesian group LASSO to identify non-linear interactions in nonparametric varying coefficient models. Ren et al. (2020) has further incorporated selection of linear and nonlinear G×E interactions simultaneously while accounting for structured identification in the Bayesian adaptive shrinkage framework. All these fully Bayesian methods can efficiently provide uncertainty quantification based on the posterior samples from MCMC. Nevertheless, our limited literature mining shows that none of the marginal Bayesian variable selection methods have been proposed for interaction studies so far.

Historically, marginal analysis has prevailed in G×E interaction studies within the framework of genetic association studies. Although recent studies have confirmed the utility of regularized variable selection in joint G×E analysis, more efforts are needed for marginal penalizations, especially through the Bayesian point of view. The step toward marginal Bayesian variable selection is of particular significance in developing a coherent framework of analyzing G×E interactions.

Here, we propose a novel marginal Bayesian variable selection method for the robust identification of G×E interactions. As heavy-tailed distributions and outliers in the response variable have been widely observed, robust modeling is essential for yielding reliable results. Specifically, the robustness of the proposed method is facilitated by the Bayesian formulation of the least absolute deviation (LAD) regression, which has been a popular choice in frequentist G×E studies but seldom investigated in a similar context from the Bayesian perspective. We consider the Bayesian LAD LASSO for regularized identification of interaction effects. As Bayesian LAD LASSO does not lead to zero coefficients, the spike-and-slab priors (George and McCulloch, 1993; Ishwaran and Rao, 2005) has been incorporated to impose exact sparsity in the adaptive shrinkage framework. The corresponding MCMC algorithm has been developed to accommodate fast computations. We have demonstrated the advantage of the proposed robust Bayesian marginal analysis in simulation. The findings from the case study of the Nurses' Health Study (NHS) with SNP measurements have important biological implications.

## 2. Method

We use *Y* to denote a continuous response variable representing the cancer outcome or disease phenotype. Let *X* = (*X*_1_, …, *X*_*p*_) be the *p* genetic variants, *E* = (*E*_1_, …, *E*_*q*_) be the *q* environmental factors and *C* = (*C*_1_, …, *C*_*m*_) be the *m* clinical factors. We denote the *i*th subject with *i*. Let (*Y*_*i*_, *E*_*i*_, *C*_*i*_, *X*_*i*_) (*i* = 1, …, *n*) be independent and identically distributed random vectors. For *X*_*ij*_ (*j* = 1, …, *p*), the measurement of the *j*th genetic factor on the *i*th subject considers the following marginal model:


(1) $$\begin{array}{l} Y_{i}=\overset{q}{\underset{k=1}{\sum }}\alpha_{k}E_{ik}+\overset{m}{\underset{t=1}{\sum }}\gamma_{t}C_{it}+\beta_{j}X_{ij}+\overset{q}{\underset{k=1}{\sum }}\eta_{jk}X_{ij}E_{ik}+\epsilon_{i}\\ \;=\overset{q}{\underset{k=1}{\sum }}\alpha_{k}E_{ik}+\overset{m}{\underset{t=1}{\sum }}\gamma_{t}C_{it}+\beta_{j}X_{ij}+\eta_{j}{\tilde{W}}_{i}+\epsilon_{i}, \end{array}$$

where α_*k*_'s and γ_*t*_'s are the regression coefficients corresponding to effects of environmental and clinical factors, respectively. For the *j*th gene *X*_*j*_ (*j* = 1, …, *p*), the G×E interactions effects are defined with *W*_*j*_ = (*X*_*j*_*E*_1_, …, *X*_*j*_*E*_*q*_), $\eta_{j}={\left(\eta_{j1},\ldots ,\eta_{jq}\right)}^{T}$. With a slight abuse of notation, denote $\tilde{W}=W_{j}$. The β_*j*_'s and η_*jk*_'s are the regression coefficients of the genetic variants and G×E interactions effects, correspondingly. Let us denote $\alpha ={\left(\alpha_{1},\ldots ,\alpha_{q}\right)}^{T}$ and $\gamma ={\left(\gamma_{1},\ldots ,\gamma_{m}\right)}^{T}$. Then model (1) can be written as:


(2) $$\begin{array}{l} Y_{i}=E_{i}\alpha +C_{i}\gamma +X_{ij}\beta_{j}+{\tilde{W}}_{i}\eta_{j}+\epsilon_{i}. \end{array}$$

### 2.1. Bayesian Formulation of the LAD Regression

The necessity of accounting for robustness in interaction studies has been increasingly recognized (Zhou et al., 2021). Within the frequentist framework, it is essentially dependent on adopting a robust loss function to quantify lack of fit (Wu and Ma, 2015). Among a variety of popular robust losses, the least absolute deviation (LAD) loss function is well known for its advantages in dealing with heavy-tailed error distributions or outliers in response. The estimation of regression coefficients amounts to the following minimization problem:


$$\begin{array}{l} \underset{\alpha ,\gamma ,\beta_{j},\eta_{j}}{\min}\overset{n}{\underset{i=1}{\sum }}|Y_{i}-E_{i}\alpha -C_{i}\gamma -X_{ij}\beta_{j}-{\tilde{W}}_{i}\eta_{j}|. \end{array}$$

Here, we propose the robust marginal Bayesian variable selection based on LAD. As the Laplace distribution is equivalent to the mixture of an exponential distribution and a scaled normal distribution (Kozumi and Kobayashi, 2011), for a Bayesian formulation of LAD regression, we assume that ϵ_*i*_(*i* = 1, …, *n*) are i.i.d. random variables following the Laplace distribution with density:


$$\begin{array}{l} f\left(\epsilon_{i}|\tau \right)=\frac{\tau }{2}\text{exp}\left(-\tau |\epsilon_{i}|\right), \end{array}$$

where τ is the inverse of the scale parameters from the Laplace density. Then the likelihood function of our marginal G×E model can be expressed as:


$$\begin{array}{l} f\left(Y|\alpha ,\gamma ,\beta_{j},\eta_{j}\right)=\overset{n}{\underset{i=1}{\prod }}\frac{\tau }{2}\text{exp}\left(-\tau |Y_{i}-E_{i}\alpha -C_{i}\gamma -X_{ij}\beta_{j}-{\tilde{W}}_{i}\eta_{j}|\right). \end{array}$$

The above formulation using Laplace distribution is a special case of the asymmetric Laplace distribution, which has been widely adopted in Baysian quantile regression (Yu and Moyeed, 2001; Yu and Zhang, 2005). In Baysian quantile regression, ϵ_*i*_'s are assumed to follow the skewed Laplace distribution with density


$$\begin{array}{l} f\left(\epsilon |\tau \right)=\theta \left(1-\theta \right)\tau \text{ exp}\left(-\tau \rho_{\theta }\left(\epsilon \right)\right), \end{array}$$

where ρ_θ_(ϵ) = ϵ{θ − *I*(ϵ < 0)} is the check loss function. The random errors can be written as


$$\begin{array}{l} \epsilon_{i}=\xi_{1}v_{i}+\tau^{-1/2}\xi_{2}\sqrt{v_{i}}z_{i}, \end{array}$$

where


$$\begin{array}{l} \xi_{1}=\frac{1-2\theta }{\theta \left(1-\theta \right)}\;and\;\xi_{2}=\sqrt{\frac{2}{\theta \left(1-\theta \right)}} \end{array}$$

with quantile level θ ∈ (0, 1), $v_{i}∽\text{exp}\left(\tau^{-1}\right)$, and *z*_*i*_∽N(0, 1).

The Bayesian LAD regression is a special case of Bayesian quantile regression (Li et al., 2010) with θ=0.5, resulting in that ξ_1_ = 0 and $\xi_{2}=\sqrt{8}$. Therefore, the response *Y*_*i*_ can be written as:


(3) $$\begin{array}{l} \;Y_{i}=\mu_{i}+\tau^{-1/2}\xi_{2}\sqrt{v_{i}}z_{i},\\ v_{i}|\tau \overset{iid}{∽}\tau \text{ exp}\left(-\tau v_{i}\right),\\ \;z_{i}\overset{iid}{∽}\text{N}\left(0,1\right), \end{array}$$

where $\mu_{i}=E_{i}\alpha +C_{i}\gamma +X_{ij}\beta_{j}+{\tilde{W}}_{i}\eta_{j}$.

### 2.2. Bayesian LAD LASSO With Spike-and-Slab Priors

In model (1), the coefficients β_*j*_ and η_*j*_ correspond to the main and interaction effects with respect to the *j*th genetic variant, respectively. When β_*j*_ = 0 and η_*j*_ = 0, the genetic variant has no effect on the phenotype. A non-zero β_*j*_ suggests the presence of main genetic effect. For η_*j*_, if at least one of its component is not zero, then the G×E interaction effect exists. In literature, Bayesian quantile LASSO, with Bayesian LAD LASSO as its special case, has been proposed to conduct variable selection (Li et al., 2010). However, a major limitation is that Bayesian quantile LASSO cannot shrink regression coefficients to 0 exactly, resulting in inaccurate identification and biased estimation. To overcome such a limitation, we incorporate spike-and-slab priors to impose sparsity within Bayesian LAD LASSO framework as follows.

For the *j*th gene (*j* = 1, …, *p*), the marginal LAD LASSO model is given by


$$\begin{array}{l} \overset{n}{\underset{i=1}{\sum }}|Y_{i}-E_{i}\alpha -C_{i}\gamma -X_{ij}\beta_{j}-{\tilde{W}}_{i}\eta_{j}|+\lambda_{1}|\beta_{j}|+\lambda_{2}\overset{q}{\underset{k=1}{\sum }}|\eta_{jk}|. \end{array}$$

Let φ_1_ = τλ_1_ and φ_2_ = τλ_2_. Then the conditional Laplace prior on the coefficient of main effect β_*j*_ can be expressed as scale mixtures of normals:


$$\begin{array}{l} \pi \left(\beta_{j}|\tau ,\lambda_{1}\right)=\frac{\varphi_{1}}{2}\text{exp}\left\{-\varphi_{1}|\beta_{j}|\right\}\\ \;=\int_{0}^{\infty }\frac{1}{\sqrt{2\pi s_{1}}}\text{exp}\left(-\frac{\beta_{j}^{2}}{2s_{1}}\right)\frac{\varphi_{1}^{2}}{2}\text{exp}\left(\frac{-\varphi_{1}^{2}}{2}s_{1}\right)ds_{1}. \end{array}$$

The conditional Laplace prior on the coefficients of interaction effect η_*j*_ can be written as:


$$\begin{array}{l} \pi \left(\eta_{j}|\tau ,\lambda_{2}\right)=\overset{q}{\underset{k=1}{\prod }}\frac{\varphi_{2}}{2}\text{exp}\left\{-\varphi_{2}|\eta_{jk}|\right\}\\ \;=\overset{q}{\underset{k=1}{\prod }}\int_{0}^{\infty }\frac{1}{\sqrt{2\pi s_{2}}}\text{exp}\left(-\frac{\eta_{jk}^{2}}{2s_{2}}\right)\frac{\varphi_{2}^{2}}{2}\text{exp}\left(\frac{-\varphi_{2}^{2}}{2}s_{2}\right)ds_{2}. \end{array}$$

Therefore, we consider the following hierarchical formulation for the marginal G×E model:


(4) $$\begin{array}{l} \;\beta_{j}|s_{1},\pi_{1}∽\left(1-\pi_{1}\right)\text{N}\left(0,s_{1}\right)+\pi_{1}\delta_{0}\left(\beta_{j}\right),\\ \;s_{1}|\varphi_{1}^{2}∽\frac{\varphi_{1}^{2}}{2}\text{exp}\left(-\frac{\varphi_{1}^{2}}{2}s_{1}\right),\\ \eta_{jk}|s_{2k},\pi_{2}\overset{iid}{∽}\left(1-\pi_{2}\right)\text{N}\left(0,s_{2k}\right)+\pi_{2}\delta_{0}\left(\eta_{jk}\right)\left(k=1,\ldots ,q\right),\\ \;s_{2k}|\varphi_{2}^{2}\overset{iid}{∽}\frac{\varphi_{2}^{2}}{2}\text{exp}\left(-\frac{\varphi_{2}^{2}}{2}s_{2k}\right)\left(k=1,\ldots ,q\right), \end{array}$$

where δ_0_(β_*j*_) and δ_0_(η_*jk*_) denote the spike at 0, respectively, and the slab distributions are represented by two normal distributions, N(0, *s*_1_) and N(0, *s*_2_*k*). Here, π_1_ ∈ [0, 1] and π_2_ ∈ [0, 1]. The mixture of the spike and slab components facilitate the selection of main and interaction effects. Instead of setting π_1_ and π_2_ to a fixed value such as 0.5, we assign conjugate beta priors on them as π_1_∽Beta(*r*_1_, *u*_1_) and π_2_∽Beta(*r*_2_, *u*_2_), which account for the uncertainty in π_1_ and π_2_. In this paper, we choose *r*_1_ = *u*_1_ = *r*_2_ = *u*_2_ = 1 as it gives a prior mean with 0.5 and it also allows a prior to spread out.

In addition, the normal prior has been placed on the coefficients of environmental factor α_*k*_(*k* = 1, …, *q*) and clinical factor γ_*t*_(*t* = 1, …, *m*) as:


$$\begin{array}{l} \alpha_{k}\overset{iid}{∽}\frac{1}{\sqrt{\left(2\pi \alpha_{0}\right)}}\text{exp}\left(-\frac{\alpha_{k}^{2}}{2\alpha_{0}}\right)\left(k=1,\ldots ,q\right)\\ \gamma_{t}\overset{iid}{∽}\frac{1}{\sqrt{\left(2\pi \gamma_{0}\right)}}\text{exp}\left(-\frac{\gamma_{t}^{2}}{2\gamma_{0}}\right)\left(t=1,\ldots ,m\right), \end{array}$$

We also assume conjugate Gamma priors on τ, $\varphi_{1}^{2}$ and $\varphi_{2}^{2}$ with


$$\begin{array}{l} \;\tau ∽\text{Gamma}\left(a,b\right),\\ \varphi_{1}^{2}∽\text{Gamma}\left(c_{1},d_{1}\right),\\ \varphi_{2}^{2}∽\text{Gamma}\left(c_{2},d_{2}\right). \end{array}$$

In typical G×E studies, the environmental and clinical factors are of low dimensionality and the selection of them is not of interest. Therefore, the sparsity-inducing priors have not been adopted for these factors. We consider the Bayesian LAD LASSO type of regularization in the proposed study as published studies have demonstrated that baseline penalty such as MCP and LASSO work well for marginal variable selection (Shi et al., 2014; Chai et al., 2017).

It is noted that Zhang et al. (2020) has proposed a marginal sparse group MCP to respect the strong hierarchy between main and interaction effects. Their results are promising when long tailed distributions and outliers are not present in the response variable. Although sparse group (or, bi-level) variable selection has been demonstrated as being very effective in multiple G×E studies based on joint models (Zhou et al., 2021), in our study, there is only one group per each marginal model. The sparse group no longer has significant advantages over individual level selection. Therefore, it has not been considered here.

Our model respects the weak hierarchy of “main effects, interactions.” If imposing the strong hierarchy is needed, the genetic factor, once it is not selected given the presence of corresponding interaction effects, can be added back to the identified marginal model for a refit to impose strong hierarchy (Chai et al., 2017). While such a practice is not uncommon in marginal interaction studies, Shi et al. (2014) has also revealed satisfactory performance when strong hierarchy has not been pursued.

### 2.3. The Gibbs Sampler for Robust Marginal G×E Analysis

For the *j*th genetic factor, the joint posterior distribution of all the unknown parameters conditional on data can be expressed as


$$\begin{array}{l} \pi (\alpha ,\gamma ,\beta_{j},\eta_{j},v,s_{1},s_{2},\tau ,\varphi_{1},\varphi_{2},\pi_{1},\pi_{2},z_{i}|Y)\\ \propto \prod_{i=1}^{n}\frac{1}{\sqrt{2\pi \tau^{-1}\xi_{2}^{2}v_{i}}}\text{exp}\left\{-\frac{{\left(y_{i}-E_{i}\alpha -C_{i}\gamma -X_{ij}\beta_{j}-{\tilde{W}}_{i}\eta_{j}\right)}^{2}}{2\tau^{-1}\xi_{2}^{2}v_{i}}\right\}\\ \times \prod_{i=1}^{n}\tau \text{ exp}(-\tau v_{i})\tau^{a-1}\text{exp}(-b\tau )\frac{1}{\sqrt{2\pi }}\text{exp}(-\frac{1}{2}z_{i}^{2})\\ \times \prod_{k=1}^{q}\frac{1}{\sqrt{\left(2\pi \alpha_{0}\right)}}\text{exp}(-\frac{\alpha_{k}^{2}}{2\alpha_{0}})\\ \times \prod_{t=1}^{m}\frac{1}{\sqrt{\left(2\pi \gamma_{0}\right)}}\text{exp}(-\frac{\gamma_{t}^{2}}{2\gamma_{0}})\\ \times \left(\left(1-\pi_{1}){\left(2\pi s_{1}\right)}^{-1/2}\text{exp}(-\frac{\beta_{j}^{2}}{2s_{1}})I_{\left\{\beta_{j}\neq 0\right\}}+\pi_{1}\delta_{0}(\beta_{j}\right)\right)\\ \times \prod_{k=1}^{q}\left(\left(1-\pi_{2}){\left(2\pi s_{2k}\right)}^{-1/2}\text{exp}(-\frac{\eta_{jk}^{2}}{2s_{2k}})I_{\left\{\eta_{jk}\neq 0\right\}}+\pi_{2}\delta_{0}(\eta_{jk}\right)\right)\\ \times \frac{\varphi_{1}^{2}}{2}\text{exp}(-\frac{\varphi_{1}^{2}}{2}s_{1})\\ \times \prod_{k=1}^{q}\frac{\varphi_{2}^{2}}{2}\text{exp}(-\frac{\varphi_{2}^{2}}{2}s_{2k})\\ \times {\left(\varphi_{1}^{2}\right)}^{c_{1}-1}\text{exp}(-d_{1}\varphi_{1}^{2})\\ \times {\left(\varphi_{2}^{2}\right)}^{c_{2}-1}\text{exp}(-d_{2}\varphi_{2}^{2})\\ \times \pi_{1}^{r_{1}-1}{\left(1-\pi_{1}\right)}^{u_{1}-1}\\ \times \pi_{2}^{r_{2}-1}{\left(1-\pi_{2}\right)}^{u_{2}-1} \end{array}$$

Let μ_(−_α__*k*_)_ = *E*(*y*_*i*_) − *E*_*ik*_α_*k*_, (*i* = 1, …, *n*), (*k* = 1, …, *q*), representing the mean effect without the contribution of *E*_*ik*_α_*k*_. The posterior distribution of the coefficient of environmental factor α_*k*_ conditional on all other parameters can be expressed as:


$$\begin{array}{l} \pi (\alpha_{k}|\text{rest})\\ \;\propto \pi (\alpha_{k})\pi (Y|\cdot )\\ \;\propto \exp\left\{-\sum_{i=1}^{n}\frac{{\left(y_{i}-E_{i}\alpha -C_{i}\gamma -X_{ij}\beta_{j}-{\tilde{W}}_{i}\eta_{j}\right)}^{2}}{2\tau^{-1}\xi_{2}^{2}v_{i}}\right\}\\ \;\times \exp(-\frac{\alpha_{k}^{2}}{2\alpha_{0}})\\ \;\propto \exp\{-\frac{1}{2}[(\sum_{i=1}^{n}\frac{\tau E_{ik}^{2}}{\xi_{2}^{2}v_{i}}+\frac{1}{\alpha_{0}})\alpha_{k}^{2}\\ \;-2\sum_{i=1}^{n}\frac{\tau (y_{i}-\mu_{\left(-\alpha_{k}\right)})E_{ik}}{\xi_{2}^{2}v_{i}}\alpha_{k}]\}. \end{array}$$

Hence, the full conditional distribution of α_*k*_ is normal distribution $N\left(\mu_{\alpha_{k}},\sigma_{\alpha_{k}}^{2}\right)$ with mean


$$\begin{array}{l} \mu_{\alpha_{k}}=(\overset{n}{\underset{i=1}{\sum }}\frac{\tau \left(y_{i}-\mu_{\left(-\alpha_{k}\right)}\right)E_{ik}}{\xi_{2}^{2}v_{i}})\sigma_{\alpha_{k}}^{2}, \end{array}$$

and variance


$$\begin{array}{l} \sigma_{\alpha_{k}}^{2}=(\overset{n}{\underset{i=1}{\sum }}\frac{\tau E_{ik}^{2}}{\xi_{2}^{2}v_{i}}+\frac{1}{\alpha_{0}}{)}^{-1}. \end{array}$$

The posterior distribution of the coefficient of clinical factor γ_*t*_(*t* = 1, …, *m*) conditional on all other parameters can be obtained in similar way. Let μ_(−_γ__*t*_)_ = *E*(*y*_*i*_) − *C*_*it*_γ_*t*_, *i* = 1, …, *n*, then


$$\begin{array}{l} \gamma_{t}|\text{rest}∽N\left(\mu_{\gamma_{k}},\sigma_{\gamma_{t}}^{2}\right), \end{array}$$

where


$$\begin{array}{l} \mu_{\gamma_{t}}=(\overset{n}{\underset{i=1}{\sum }}\frac{\tau \left(y_{i}-\mu_{\left(-\gamma_{t}\right)}\right)C_{it}}{\xi_{2}^{2}v_{i}})\sigma_{\gamma_{t}}^{2},\\ \sigma_{\gamma_{t}}^{2}=(\overset{n}{\underset{i=1}{\sum }}\frac{\tau C_{it}^{2}}{\xi_{2}^{2}v_{i}}+\frac{1}{\gamma_{0}}{)}^{-1}. \end{array}$$

Let μ_(−_β__*j*_)_ = *E*(*y*_*i*_) − *X*_*ij*_β_*j*_ and *l*_1_ = π(β_*j*_ = 0|rest), the conditional posterior distribution of the coefficient of genetic factor β_*j*_ is a spike-and-slab distribution:


(5) $$\begin{array}{l} \beta_{j}|\text{rest}∽\left(1-l_{1}\right)N\left(\mu_{\beta_{j}},\sigma_{\beta_{j}}^{2}\right)+l_{1}\delta_{0}\left(\beta_{j}\right), \end{array}$$

where


$$\begin{array}{l} \mu_{\beta_{j}}=(\overset{n}{\underset{i=1}{\sum }}\frac{\tau \left(y_{i}-\mu_{\left(-\beta_{j}\right)}\right)X_{ij}}{\xi_{2}^{2}v_{i}})\sigma_{\beta_{j}}^{2},\\ \sigma_{\beta_{j}}^{2}=(\overset{n}{\underset{i=1}{\sum }}\frac{\tau X_{ij}^{2}}{\xi_{2}^{2}v_{i}}+\frac{1}{s_{1}}{)}^{-1}. \end{array}$$

We can show that


$$\begin{array}{l} l_{1}=\frac{\pi_{1}}{\pi_{1}+\left(1-\pi_{1}\right)s_{1}^{-1/2}{\left(\sigma_{\beta_{j}}^{2}\right)}^{1/2}\text{exp}\left\{\frac{1}{2}{\left(\sum_{i=1}^{n}\frac{\tau \left(y_{i}-\mu_{\left(-\beta_{j}\right)}\right)X_{ij}}{\xi_{2}^{2}v_{i}}\right)}^{2}\sigma_{\beta_{j}}^{2}\right\}}. \end{array}$$

The posterior distribution of β_*j*_ is a mixture of a normal distribution and a point mass at 0. That is, at each iteration of MCMC, β_*j*_ is drawn from $N\left(\mu_{\beta_{j}},\sigma_{\beta_{j}}^{2}\right)$ with probability (1−*l*_1_) and is set to 0 with probability *l*_1_.

Similarly, the posterior distribution of the interaction of the *j*th gene and environmental factors η_*jk*_(*k* = 1, …, *q*) is also a spike-and-slab distribution. Denote μ_(−_η__*jk*_)_ = *E*(*y*_*i*_) − *W*_*ik*_η_*jk*_ and *l*_2*k*_ = π(η_*jk*_ = 0|rest), η_*jk*_ follows this distribution:


(6) $$\begin{array}{l} \eta_{jk}|\text{rest}∽\left(1-l_{2k}\right)N\left(\mu_{\eta_{jk}},\sigma_{\eta_{jk}}^{2}\right)+l_{2k}\delta_{0}\left(\eta_{jk}\right), \end{array}$$

where


$$\begin{array}{l} \mu_{\eta_{jk}}=(\overset{n}{\underset{i=1}{\sum }}\frac{\tau \left(y_{i}-\mu_{\left(-\eta_{jk}\right)}\right){\tilde{W}}_{ik}}{\xi_{2}^{2}v_{i}})\sigma_{\eta_{jk}}^{2},\\ \sigma_{\beta_{j}}^{2}=(\overset{n}{\underset{i=1}{\sum }}\frac{\tau {\tilde{W}}_{ik}^{2}}{\xi_{2}^{2}v_{i}}+\frac{1}{s_{2k}}{)}^{-1}. \end{array}$$

And


(7) $$\begin{array}{l} l_{2k}=\frac{\pi_{2}}{\pi_{2}+\left(1-\pi_{2}\right)s_{2k}^{-1/2}{\left(\sigma_{\eta_{jk}}^{2}\right)}^{1/2}\text{exp}\left\{\frac{1}{2}{\left(\sum_{i=1}^{n}\frac{\tau \left(y_{i}-\mu_{\left(-\eta_{jk}\right)}\right){\tilde{W}}_{ik}}{\xi_{2}^{2}v_{i}}\right)}^{2}\sigma_{\eta_{jk}}^{2}\right\}}. \end{array}$$

The full conditional posterior distribution of *s*_1_ is:


(8) $$\begin{array}{l} s_{1}|\text{rest}\\ \;\propto \pi \left(\beta_{j}|s_{1},\pi_{1}\right)\pi \left(s_{1}|\varphi_{1}^{2}\right)\\ \;\propto (\left(1-\pi_{1}\right){\left(2\pi s_{1}\right)}^{-1/2}\text{exp}\left(-\frac{\beta_{j}^{2}}{2s_{1}}\right){\text{I}}_{\left\{\beta_{j}\neq 0\right\}}\\ \;+\pi_{1}\delta_{0}\left(\beta_{j}\right))\text{exp}\left(-\frac{\varphi_{1}^{2}}{2}s_{1}\right). \end{array}$$

When β_*j*_ = 0, equation (8) is proportional to $\text{exp}\left(-\frac{\varphi_{1}^{2}}{2}s_{1}\right)$. Therefore, the posterior distribution of *s*_1_ is $\text{exp}\left(\frac{\varphi_{1}^{2}}{2}\right)$.

When β_*j*_ ≠ 0, equation (8) is proportional to


$$\begin{array}{l} \frac{1}{\sqrt{s_{1}}}\text{exp}(-\frac{\varphi_{1}^{2}}{2}s_{1})\text{exp}(-\frac{\beta_{j}^{2}}{2s_{1}})\\ \;\propto \frac{1}{\sqrt{s_{1}}}\text{exp}\left\{-\frac{1}{2}[\varphi_{1}^{2}s_{1}+\frac{\beta_{j}^{2}}{s_{1}}]\right\}. \end{array}$$

Therefore, when β_*j*_ ≠ 0, the posterior distribution for $s_{1}^{-1}$ is Inverse-Gaussian$\left(\sqrt{\frac{\varphi_{1}^{2}}{\beta_{j}^{2}}},\varphi_{1}^{2}\right)$.

Similarly, for *s*_2*k*_(*k* = 1, …, *q*), when η_*jk*_ = 0, the posterior distribution of *s*_2*k*_ is $\text{exp}\left(\frac{\varphi_{2}^{2}}{2}\right)$. When η_*jk*_ ≠ 0, the posterior distribution for $s_{2k}^{-1}$ is Inverse-Gaussian$\left(\sqrt{\frac{\varphi_{2}^{2}}{\eta_{jk}^{2}}},\varphi_{2}^{2}\right)$.

The full conditional posterior distribution of $\varphi_{1}^{2}$:


$$\begin{array}{l} \varphi_{1}^{2}|\text{rest}\\ \;\propto \pi \left(s_{1}|\varphi_{1}^{2}\right)\pi \left(\varphi_{1}^{2}\right)\\ \;\propto \frac{\varphi_{1}^{2}}{2}\text{exp}\left(-\frac{\varphi_{1}^{2}s_{1}}{2}\right){\left(\varphi_{1}^{2}\right)}^{c_{1}-1}\text{exp}\left(-d_{1}\varphi_{1}^{2}\right)\\ \;\propto {\left(\varphi_{1}^{2}\right)}^{c_{1}}\text{exp}\left(-\varphi_{1}^{2}\left(s_{1}/2+d_{1}\right)\right). \end{array}$$

Therefore, the posterior distribution for $\varphi_{1}^{2}$ is Gamma (*c*_1_+1, *s*_1_/2+*d*_1_). Similarly, the posterior distribution for $\varphi_{2}^{2}$ is Gamma ($c_{2}+q,\sum_{k=1}^{q}s_{2k}/2+d_{2}$).

The full conditional posterior distribution of π_1_ is given as:


$$\begin{array}{l} \pi_{1}|\text{rest}\\ \;\propto \pi \left(s_{1}|\varphi_{1}^{2}\right)\pi \left(\varphi_{1}^{2}\right)\\ \;\propto \pi_{1}^{r_{1}-1}{\left(1-\pi_{1}\right)}^{u_{1}-1}\\ \;\times \left(\left(1-\pi_{1}\right){\left(2\pi s_{1}\right)}^{-1/2}\text{exp}\left(-\frac{\beta_{j}^{2}}{2s_{1}}\right){\text{I}}_{\left\{\beta_{j}\neq 0\right\}}+\pi_{1}\delta_{0}\left(\beta_{j}\right)\right). \end{array}$$

Then, the posterior distribution for π_1_ is Beta (1 + *r*_1_ − **I**(β_*j*_ ≠ 0), *u*_1_ + **I**(β_*j*_ ≠ 0)).

The full conditional posterior distribution of π_2_ is given as:


$$\begin{array}{l} \pi_{2}|\text{rest}\\ \;\propto \pi \left(s_{2}|\varphi_{2}^{2}\right)\pi \left(\varphi_{2}^{2}\right)\\ \;\propto \pi_{2}^{r_{2}-1}{\left(1-\pi_{2}\right)}^{u_{2}-1}\\ \;\times \overset{q}{\underset{k=1}{\prod }}\left(\left(1-\pi_{2}\right){\left(2\pi s_{2k}\right)}^{-1/2}\text{exp}\left(-\frac{\eta_{jk}^{2}}{2s_{2k}}\right){\text{I}}_{\left\{\eta_{jk}\neq 0\right\}}+\pi_{2}\delta_{0}\left(\eta_{jk}\right)\right). \end{array}$$

So, the posterior distribution for π_2_ is Beta $\left(1+r_{1}-\sum_{k=1}^{q}\text{I}\left(\eta_{jk}\neq 0\right),u_{1}+\sum_{k=1}^{q}\text{I}\left(\eta_{jk}\neq 0\right)\right)$.

The full conditional posterior distribution of τ is given as:


$$\begin{array}{l} \tau |\text{rest}\\ \;\propto \pi (v|\tau )\pi (\tau )\pi (Y|\cdot )\\ \;\propto \tau^{n/2}\exp\left\{-\sum_{i=1}^{n}\frac{{\left(y_{i}-E_{i}\alpha -C_{i}\gamma -X_{ij}\beta_{j}-{\tilde{W}}_{i}\eta_{j}\right)}^{2}}{2\tau^{-1}\xi_{2}^{2}v_{i}}\right\}\\ \;\times \;\tau^{n}\text{exp}(-\tau \sum_{i=1}^{n}v_{i})\tau^{a-1}\text{exp}(-b\tau )\\ \;\propto \tau^{a+\frac{3}{2}n-1}\text{exp}\{-\tau [\sum_{i=1}^{n}(\frac{{\left(y_{i}-E_{i}\alpha -C_{i}\gamma -X_{ij}\beta_{j}-{\tilde{W}}_{i}\eta_{j}\right)}^{2}}{2\xi_{2}^{2}v_{i}}\\ \;+v_{i})+b]\}. \end{array}$$

Therefore, the posterior distribution for τ is Gamma($a+\frac{3}{2}n,[\sum_{i=1}^{n}\left(\frac{{\left(y_{i}-E_{i}\alpha -C_{i}\gamma -X_{ij}\beta_{j}-{\tilde{W}}_{i}\eta_{j}\right)}^{2}}{2\xi_{2}^{2}v_{i}}+v_{i}\right)+b]$).

Last, we have the full conditional posterior distribution of *v*_*i*_:


$$\begin{array}{l} v_{i}|\text{rest}\\ \;\propto \pi \left(v|\tau \right)\pi \left(Y|\cdot \right)\\ \;\propto \frac{1}{\sqrt{v_{i}}}\exp\left\{-\frac{{\left(y_{i}-E_{i}\alpha -C_{i}\gamma -X_{ij}\beta_{j}-{\tilde{W}}_{i}\eta_{j}\right)}^{2}}{2\tau^{-1}\xi_{2}^{2}v_{i}}\right\}\\ \;\times \exp\left(-\tau v_{i}\right)\\ \;\propto \frac{1}{\sqrt{v_{i}}}\exp\{-\frac{1}{2}[\left(2\tau \right)v_{i}\\ \;+\frac{\tau {\left(y_{i}-E_{i}\alpha -C_{i}\gamma -X_{ij}\beta_{j}-{\tilde{W}}_{i}\eta_{j}\right)}^{2}}{\xi_{2}^{2}v_{i}}]\}. \end{array}$$

It is easy to show that


$$\begin{array}{l} \frac{1}{v_{i}}|\text{rest}∽\text{Inverse-Gaussian}\\ \left(\sqrt{\frac{2\xi_{2}^{2}}{{\left(y_{i}-E_{i}\alpha -C_{i}\gamma -X_{ij}\beta_{j}-{\tilde{W}}_{i}\eta_{j}\right)}^{2}}},2\tau \right). \end{array}$$

The spirit of marginal penalization for G×E interactions lies in the usage of a common sparsity cutoff to determine a list of important main and interaction effects. Instead of focusing on a fixed cutoff, varying the cutoff can generate different lists, resulting in a comprehensive view of important findings. The tuning parameter in penalized estimation serves as the cutoff. Therefore, the same tuning parameter has to be adopted for all the sub-models (Shi et al., 2014; Chai et al., 2017; Zhang et al., 2020). To further justify such a common tuning parameter, Zhang et al. (2020) has attempted using the joint model to select the common tuning through cross-validation. However, this seems not coherent with the nature of marginal analysis.

Ideally, the tuning parameter should be determined by each model itself to allow for flexibility in controlling sparsity individually, and a common cutoff is still available to examine different lists of important effects. With the Bayesian formulation, we can avoid such a limitation of frequentist marginal penalization methods. In particular, the priors have been placed on regularization parameters to determine the sparsity in a data-driven manner for each sub-model. With the spike-and-slab priors, the posterior distributions on the coefficients of main and interaction effects naturally lead to the usage of inclusion probability as a common cutoff to pin down the list of important effects, which is described in detail in the next section.

## 3. Simulation

To demonstrate the utility of the proposed approach, we evaluate the performance through simulation study. In particular, we compare the performance of the proposed method, LAD Bayesian Lasso with spike-and-slab priors (denoted as LADBLSS) with three alternatives, LAD Bayesian Lasso (denoted as LADBL), Bayesian Lasso with spike-and-slab priors (denoted as BLSS) and Bayesian Lasso (denoted as BL). LADBL is similar to the proposed method, except that it does not adopt the spike-and-slab prior. The details of posterior inference are given in the Appendix.

Under all settings, the sample size is set as *n* = 200, and the number of G factors is *p* = 500 with *q* = 4, *m* = 3. For environmental factors, we simulate four continuous variables from multivariate normal distributions with marginal mean 0, marginal variance 1 and AR1 correlation structure with ρ = 0.5. In addition, three clinical factors are generated from a multivariate normal distribution with marginal mean 0 and marginal variance 1 and AR1 structure with ρ = 0.5. Among the *p* main G effects and *pq* G×E interactions, 8 and 12 effects are set as being associated with the response, respectively. All the environmental and clinical factors are important with nonzero coefficients, which are randomly generated from a uniform distribution Unif[0.1, 0.5]. The random error are generated from: (1) N(0, 1)(Error 1), (2) t-distribution with 2 degrees of freedom (*t*(2)) (Error2), (3) LogNormal(0,2)(Error3), (4) 90%N(0,1)+10%Cauchy(0,1)(Error4), (5) 80%N(0,1)+20%Cauchy(0,1)(Error5). All of them are heavy-tailed distribution except the first one.

In addition, the genetic factors are simulated in the following four settings.

*Setting 1*: In simulating continuous genetic variants, we generate multivariate normal distributions with marginal mean 0 and variance 1. The AR structure is considered in computing the correlation of G factors, under which gene *j* and *k* have correlation ρ^|*j*−*k*|^ with ρ = 0.5.

*Setting 2*: We assess the performance under single-nucleotide polymorphism (SNP) data. The SNPs are obtained by dichotomizing the gene expression values at the 1st and 3rd quartiles, with the 3 levels (0,1,2) for genotypes (aa, Aa, and AA). Here, the gene expressions are generated from the first setting.

*Setting 3*: Consider simulating the SNP data under a pairwise linkage disequilibrium (LD) structure. For the two minor alleles A and B of two adjacent SNPs, let *q*_1_ and *q*_2_ be the minor allele frequencies (MAFs). The frequencies of four haplotypes are as *p*_*AB*_ = *q*_1_*q*_2_ + δ, *p*_*ab*_ = (1 − *q*_1_)(1 − *q*_2_) + δ, *p*_*Ab*_ = *q*_1_(1 − *q*_2_) − δ, and *p*_*aB*_ = (1 − *q*_1_)*q*_2_ − δ, where δ denotes the LD. Assuming Hardy–Weinberg equilibrium and given the allele frequency for A at locus 1, we can generate the SNP genotype (AA, Aa, aa) from a multinomial distribution with frequencies $\left(q_{1}^{2},2q_{1}\left(1-q_{1}\right),{\left(1-q_{1}\right)}^{2}\right)$. Based on the conditional genotype probability matrix, we can simulate the genotypes for locus 2. With MAFs 0.3 and pairwise correlation *r* = 0.6, we have $\delta =r\sqrt{q_{1}\left(1-q_{1}\right)q_{2}\left(1-q_{2}\right)}$.

We collect the posterior samples from the Gibbs Sampler with 10,000 iterations and discard the first 5,000 samples as burn-ins. The posterior medians are used to estimate the coefficients. For approaches incorporating spike-and-slab priors, we consider computing the inclusion probability to indicate the importance of predictors. Here, we use a binary indicator ϕ to denote that the membership of the non-spike distribution. Take the main effect of the *j*th genetic factor, *X*_*j*_, as an example. Suppose we have collected H posterior samples from MCMC after burn-ins. The *j*th G factor is included in the marginal G×E model at the *h*th MCMC iteration if the corresponding indicator is 1, i.e., $\phi_{j}^{\left(h\right)}=1$. Subsequently, the posterior probability of retaining the *j*th genetic main effect in the final marginal model is defined as the average of all the indicators for the *j*th G factor among the H posterior samples. That is,


$$\begin{array}{l} p_{j}=\hat{\pi }\left(\phi_{j}=1|y\right)=\frac{1}{H}\overset{H}{\underset{h=1}{\sum }}\phi_{j}^{\left(h\right)},j=1,\ldots ,p. \end{array}$$

A larger posterior inclusion probability *p*_*j*_ indicates a stronger empirical evidence that the *j*th genetic main effect has a non-zero coefficient, i.e., a stronger association with the phenotypic trait.

To comprehensively assess the performance of the proposed and alternative methods, we consider a sequence of probabilities as cutting-offs in inclusion probability for methods with spike-and-slab priors. Given a cutoff probability, the main or interaction is included in the final marginal model if its posterior inclusion probability is larger than the cutoff, and is excluded otherwise. Provided with a sequence of cutting-off probabilities from small to large, we can investigate the set of identified effects and calculate the true/false positive rates (T/FPR) as the ground truth is known in simulation. For the sequence of cut-offs, we are able to compute the area under curve (AUC) as a comprehensive measure. Besides, for methods without spike-and-slab priors, the confidence level of the credible intervals can be adopted as the cut-off to compute TPR and FPRs. Therefore, all the methods under comparison can be evaluated on the same ground.

In addition, we also consider Top100, which is defined as the number of true signals when 100 important main effects (or interactions) are identified. For methods with spike-and-slab priors, 100 main effects or interactions are chosen with the highest inclusion probabilities. For methods without spike-and-slab priors, the indicators of all effects are computed for a sequence of credible levels. The top 100 main effects or interactions are chosen in terms of the highest average identification values.

Simulation results for the gene expression data in the first setting are tabulated in Tables 1, 2. We can observe that the proposed method has the best performance among all approaches, especially when the response variable has heavy-tailed distributions. First, the performance of methods with spike-and-slab priors is consistently better than methods without spike-and-slab priors. For example, in Table 1, under error 3, the AUC of LADBLSS is 0.9558 (sd 0.0161), which is much larger than that of the robust method without spike-and-slab priors, i.e., 0.8432(sd 0.0115) from LADBL. Also, the AUC of robust methods is much larger than that of non-robust methods, especially in the presence of heavy-tailed errors. For instance, in the first setting under error 3, the AUC of LADBLSS is 0.9558 and the AUC of LADBL is 0.8432 while that of BLSS and BL is around 0.5. Similar advantageous performance can also be observed from the identification results with Top100. In Table 2 under error 5, LADBLSS identifies 7.80 (sd 0.55) out of the 8 main effects and 10.53 (sd 1.36) out of the 12 interaction effects. This is higher than the results of LADBL with 7.57 (sd 0.57) of main effects and 6.83 (sd 1.07) of interaction effects. Second, among all the methods with spike-and-slab priors, Bayesian LAD method with spike-and-slab priors has the best performance in all identification results. Under error 3, in Table 1, the AUC of LADBLSS is 0.9558(sd 0.0161) while the AUC of BLSS is 0.5473(sd 0.0576). Under error 4 in Table 2, LADBLSS identifies 7.77(sd 0.57) main effects and 10.67(sd 1.50) interaction effects while BLSS identifies 6.2(sd 2.62) main effects and 8.3(sd 3.98) interaction effects, respectively.

**Table 1:** Simulation results of the first setting for BL (Bayesian LASSO), BLSS (Bayesian LASSO with spike-and-slab priors), LADBL (LAD Bayesian LASSO), and LADBLSS (LAD Bayesian LASSO with spike-and-slab priors).

		**BL**	**BLSS**	**LADBL**	**LADBLSS**
Error 1	AUC	0.9182	0.9901	0.9258	0.9887
N(0,1)	SD	0.0052	0.0021	0.0076	0.0026
Error 2	AUC	0.8332	0.9420	0.9004	0.9841
*t*(2)	SD	0.0107	0.0235	0.0078	0.0031
Error 3	AUC	0.5343	0.5473	0.8432	0.9558
Lognormal(0,2)	SD	0.0144	0.0576	0.0115	0.0161
Error 4	AUC	0.8221	0.9124	0.9222	0.9895
90%N(0,1) + 10%Cauchy(0,1)	SD	0.0212	0.0410	0.0071	0.0024
Error 5	AUC	0.7507	0.8431	0.9192	0.9904
80%N(0,1) + 20%Cauchy(0,1)	SD	0.0217	0.0633	0.0059	0.0018

*AUC (mean of AUC) and SD (sd of AUC) based on 100 replicates. n = 200, p = 500, q = 4, and m = 3*.

**Table 2:** Identification results of the first setting with Top100 method for BL (Bayesian LASSO), BLSS (Bayesian LASSO with spike-and-slab priors), LADBL (LAD Bayesian LASSO) and LADBLSS (LAD Bayesian LASSO with spike-and-slab priors).

		**Main**	**Interaction**	**Total**
Error 1	BL	7.60(0.49)	6.80(1.6)	14.40(1.73)
N(0,1)	BLSS	7.80(0.41)	10.80(0.92)	18.60(1.13)
	LADBL	7.67(0.55)	6.53(1.85)	14.20(1.81)
	LADBLSS	7.76(0.5)	10.53(1.36)	18.30(1.49)
Error 2	BL	6.37(1.90)	3.90(2.07)	10.27(3.19)
*t*(2)	BLSS	6.33(1.63)	8.53(2.46)	14.87(3.71)
	LADBL	7.43(0.94)	5.80(1.71)	13.23(2.01)
	LADBLSS	7.53(0.51)	9.90(1.56)	17.43(1.76)
Error 3	BL	0.90(1.21)	0.50(0.97)	1.40(1.45)
Lognormal(0,2)	BLSS	0.73(0.94)	0.47(0.68)	1.20(1.35)
	LADBL	6.27(1.55)	3.67(1.94)	9.93(2.75)
	LADBLSS	6.10(1.37)	8.93(2.02)	15.03(3.09)
Error 4	BL	5.57(2.99)	3.63(2.53)	9.20(5.05)
90%N(0,1)	BLSS	6.20(2.62)	8.30(3.98)	14.50(6.39)
+10%Cauchy(0,1)	LADBL	7.77(0.43)	7.00(1.93)	14.77(1.81)
	LADBLSS	7.77(0.57)	10.67(1.50)	18.23(1.67)
Error 5	BL	5.07(2.89)	3.00(2.49)	8.07(5.01)
80%N(0,1)	BLSS	4.60(3.25)	5.70(4.23)	10.30(7.27)
+20%Cauchy(0,1)	LADBL	7.57(0.57)	6.83(1.07)	14.40(1.83)
	LADBLSS	7.80(0.55)	10.53(1.36)	18.33(1.69)

*Mean(sd) based on 100 replicates. n = 200, p = 500, q = 4, and m = 3*.

**Figure 1:**
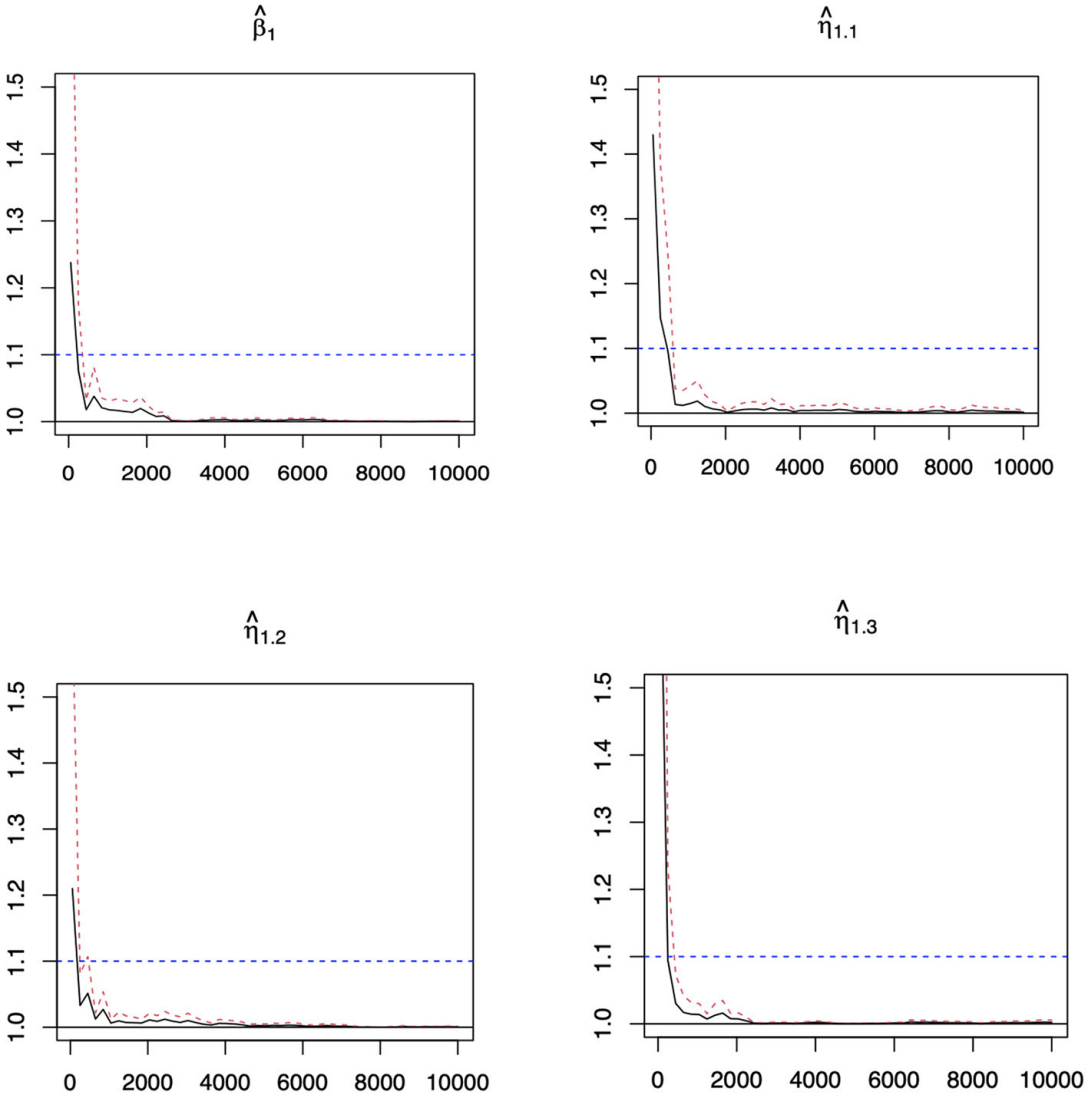
Potential scale reduction factor (PSRF) against iterations for the coefficients of the first genetic factors and its interaction with environmental factors in Example 1 under error 3. Black line: the PSRF. Red dotted line: the upper limits of the 95% confidence interval for the PSRF. Blue dotted line: The threshold of 1.1. The ${\hat{\beta }}_{1}$ represents the estimated coefficients of the main effects for the first genetic factor. The ${\hat{\eta }}_{11}$ to ${\hat{\eta }}_{13}$ represent the estimated coefficients of the first three interaction effects for the first genetic factor.

Similar patterns can be observed in Tables 4, 5 in Appendix for the second setting, and Tables 6, 7 in Appendix for the third setting in Appendix. We have also investigated the performance of when *n* = 2,000 under setting 1. While the difference among the 4 methods significantly diminishes with such a large sample size, we can still observe the superior performance of LADBLSS by using a shorter list of top ranked effects. The results are provided in the table from Supplementary Material. Overall, the advantages of conducting robust Bayesian G×E analysis using the proposed approach can be justified based on the results of comprehensive simulation studies. The convergence of the MCMC chains with the potential scale reduction factor (PSRF) (Brooks and Gelman, 1998) has been conducted. In this study, we use PSRF ≤ 1.1 (Gelman et al., 2004) as the cut-off point, which indicates that chains converge to a stationary distribution. The convergence of chains after burn-ins has been checked for all parameters with the value of PSRF < 1.1. Figure 1 shows the convergence pattern of PSRF for the main and interaction coefficients of the first genetic factors in Example 1 under error 3.

In simulation, the hyperparameters for the Gamma priors and Beta priors specified in section Bayesian LAD LASSO With Spike-and-slab Priors are set to 1. In addition, the initial values of the regression parameters are also set to 1. Based on our experiments, the results and convergence of the MCMC algorithm are not sensitive to the choice of these parameters. We have observed satisfactory convergence for all of our simulations. For one simulated dataset under the first setting with *n* = 200, *p* = 500 and standard normal error, the CPU time (in minutes) for fitting all the 500 marginal models through 10,000 MCMC iterations on a laptop with standard configurations are 1.27(BL), 1.75(BLSS), 6.16(LADBL), and 5.95 (LADBLSS) minutes, respectively. The source codes of implementing all the methods under comparison are included in the Supplementary Material.

## 4. Real Data Analysis

In this study, we analyze the type 2 diabetes (T2D) data from Nurses' Health Study (NHS), which is a well-characterized cohort study of women with high-dimensional SNP data, as well as measurements on lifestyle and dietary factors. We consider SNPs on chromosome 10 to identify main and gene–environment interactions associated with weight, which is an important phenotypic trait related to type 2 diabetes. Here, weight is used as response and five environment factors, age (age), total physical activity (act), trans fat intake (trans), cereal fiber intake (ceraf), and reported high blood cholesterol (chol), are considered. Data are available on 3,391 subjects and 17,016 gene expressions after cleaning the raw data through matching phenotypes and genotypes and removing SNPs with MAF <0.05. A prescreening is done before downstream analysis. We use a marginal linear model with weight as response and age, act, trans, ceraf, and chol as environment factors. Note that 10,000 SNPs that have at least two main or interaction effects with *p* < 0.05 are kept.The scale of working data is generally not a major concern for marginal analysis, as the computation can be done in a highly parallel manner. Here, we focus on chromosome 10 which has been reported to harbor interesting genes in existing studies.

We use Top 100 method to identify 100 most important main and interaction effects. The proposed method LADBLSS identifies 20 main SNP effects and 80 gene–environment interactions, which are listed in Table 8 in Appendix. Our study provides crucial implications in identifying the important main and interactions of SNPs and its associations with weight. For example, three SNPs, rs17011106, rs4838643 and rs17011115, located within gene WDFY4 are identified. WDFY4 has been observed as an influential factor related to weight and obesity (Barclay et al., 2015; Martin et al., 2019). In addition, SNPs rs10994364, rs10821773, and rs10994308, located within gene ANK3, are identified with interacting environment factors age and chol. There are findings showing an association between ANK3 and higher systolic blood pressure (Ghanbari et al., 2014). Published studies have also shown that ANK3 is linked to pulmonary and renal hypertension (Ghanbari et al., 2014). Allele risk variants have been identified in ANK3, and these variants explain a proportion of the heritability of BD (bipolar disorder), which is associated with higher body mass index (BMI) and increased metabolic comorbidity and the genetic risk for BD relates to common genetic risk with T2D (Winham et al., 2014). Our proposed method identifies its interaction with chol, the high blood cholesterol. Data from several sources suggest that islet cholesterol metabolism contributes to the pathogenesis of T2D (Brunham et al., 2008). Furthermore, the SNP rs1244416, corresponding to gene ATP5C1, interacts with the reported high blood cholesterol. This gene has been found to be deregulated in T2D skeletal muscle through pathway-based microanalysis (Morrison et al., 2012). The interactions between SNP rs10857590 and trans fat intake has also been identified by using the proposed method. The SNP is within gene ARHGAP22, which has been investigated in Huang et ail. (2018). As a diabetic retinopathy (DR) susceptibility gene, the expression of ARHGAP22 is positively associated with endothelial progenitor cells (EPC) levels in T2D patients with DR.

Analysis with alternatives BL, BLSS, and LADBL has also been conducted. To compare the alternative methods with the proposed method, we provide the numbers of main effects and interactions identified by these methods with pairwise overlaps in Table 3. It clearly shows that the proposed one results in a very different set of effects compared to alternatives. We refit the regularized marginal models by LADBL and LADBLSS using robust Bayesian Lasso, and those identified by BL and BLSS using Bayesian Lasso. In addition, the inclusion probabilities of the selected main and interaction effects using LADBLSS are provided in Table 9 in Appendix. Results from the alternative methods are available from the Supplementary Material. The proposed method selects the 100 most important effects with the inclusion probability larger than 0.9, demonstrating its superiority in quantifying uncertain compared to marginal penalization methods (Shi et al., 2014; Chai et al., 2017; Zhang et al., 2020). We noticed the small magnitude of refitted regression coefficients from LAD-based methods compared to those obtained by the non-robust method in the Supplementary Material. This is due to the difference between the LAD-based and least square based loss function for robust and non-robust methods, respectively. The advantage of LADBLSS over the non-robust methods can be clearly observed. First, majority of the top 100 important effects identified by BL are main genetic effects. This is less likely to be reasonable as the response variable weight has been well acknowledged to be also dependent on gene–environment interactions. For BLSS, the inclusion probabilities are low compared to those of the LADBLSS, suggesting lower level of certainty and confidence in the regression coefficients obtained from BLSS. The inferior performance of BL and BLSS further justifies the need of developing robust methods in marginal gene–environment interaction studies. Overall, LADBLSS leads to identification results significantly different from all the alternatives, as well as main and interaction effects of important biological implications that are not discovered by the benchmarks.

**Table 3:** The numbers of main G effects and interactions identified by different approaches and their overlaps for BL (Bayesian LASSO), BLSS (Bayesian LASSO with spike-and-slab priors), LADBL (LAD Bayesian LASSO), and LADBLSS (LAD Bayesian LASSO with spike-and-slab priors).

**T2D**	**Main**				**Interaction**			
	**BL**	**BLSS**	**LADBL**	**LADBLSS**	**BL**	**BLSS**	**LADBL**	**LADBLSS**
BL	86	5	6	8	14	14	4	8
BLSS		24	3	6		76	20	23
LADBL			20	12			80	50
LADBLSS				20				80

## 5. Discussion

In the past, G×E interaction studies have been mainly conducted through marginal hypothesis testing, based on a diversity of study designs utilizing parametric, nonparametric, and semiparametric models (Murcray et al., 2009; Thomas, 2010; Mukherjee et al., 2012), which later have been extended to joint analyses driven primarily by the pathway or gene set based association studies (Wu and Cui, 2013a; Jin et al., 2014; Jiang et al., 2017). In addition, published literature has also reported the success of marginal screening studies, including those based on partial correlations (Niu et al., 2018; Xu et al., 2019). Recently, the effectiveness of regularized variable selection in G×E interaction studies has been increasingly recognized, and a large number of regularization methods have been proposed for joint interaction studies (Zhou et al., 2021). Marginal penalization has also been demonstrated as promising competitors, although they have only been investigated in a limited number of frequentist studies (Shi et al., 2014; Chai et al., 2017; Zhang et al., 2020).

Therefore, the proposed marginal robust Bayesian variable selection is of particular importance, since joint and marginal analysis cannot replace each other and marginal Bayesian penalization has not been examined for G×E studies so far. In particular, with the robustness and incorporation of spike-and-slab priors in the adaptive Bayesian shrinkage, the LADBLSS has an analysis framework more coherent with that of the joint robust analysis^1^, which significantly facilitates methodological developments for interaction studies.

Nevertheless, the proposed method has limitations. As a fully Bayesian methods based on MCMC algorithms, the computation cost is generally high due to the tradeoff for quantifying uncertainty using posterior samples. Such a drawback can be addressed through conducting the computation in a parallel manner given the marginal nature of the method. Besides, the variable selection conducted in our study is based on the L1 penalty within the Bayesian framework. As this structure ignores the correlation among genetic features, a possible direction for future improvement is to incorporate network or gene set information in the identification of important gene–environment interactions (Wang et al., 2021). Furthermore, in our study, the genetic factor is represented by one SNP coded as a triadic factor. A closer look at both the additive and dominant penetrance effects of the SNP will lead to elucidation of the genetic basis using marginal interaction studies on a finer scale. For gene–environment interaction studies, marginal and joint analysis are the two major paradigms, and cannot replace each other (Zhou et al., 2021). It is always on a safe side to perform marginal analysis in G×E studies in addition to the joint ones, facilitating a more comprehensive understanding on the genetic architecture of complex diseases.

The marginal Bayesian regularization can be extended to different types of response, for example, under binary, categorical, prognostic and multivariate outcomes. Nevertheless, considering robustness in the generalized models with the Bayesian framework is not trivial, especially under the multivariate responses (Wu et al., 2014; Zhou et al., 2019). We postpone the investigations to the future studies.The interaction between genetic and environmental factors in this study has been modeled as the product of the two corresponding variables, which amounts to “linear” interactions. In practice, the linear interaction assumption has been frequently violated (Ma et al., 2011; Wu and Cui, 2013b; Zhao et al., 2019), which demands accommodation of these nonlinear effects through nonparametric and semiparametric models (Li et al., 2015; Wu et al., 2015, 2018; Ren et al., 2020). It is of great interest and importance to migrate the nonlinear G×E studies to marginal cases in the near future.

## Data Availability Statement

Publicly available datasets were analyzed in this study. This data can be found here: Authorized access should be granted before accessing the data. Applications to access the data should be sent to dbGap (accession number phs000091.v2.p1). For more information, please refer to NIH dbGap (https://www.ncbi.nlm.nih.gov/projects/gap/cgi-bin/study.cgi?study_id=phs000091.v2.p1).

## Ethics Statement

The studies involving human participants were reviewed and approved by This study is a secondary data analysis. The dataset has been applied through NIH dbGap (https://www.ncbi.nlm.nih.gov/projects/gap/cgi-bin/study.cgi?study_id=phs000091.v2.p1). In the dataset, the patient information has been de-identified. As indicated from the dbGap website under section Authorized Access/Use Restrictions, IRB is not required for accessing and using the data. According to the original publication, The study was approved by the institutional review board of Brigham and Women's Hospital in Boston; completion of the self-administered questionnaire was considered to imply informed consent. For more information regarding study population, please refer to the original publication: Hu et al. (2001). The patients/participants provided their written informed consent to participate in this study.

## Author Contributions

XL and CW: conceptualization and writing—original draft preparation. XL, KF, JR, and CW: methodology and writing—review and editing. XL: data analysis. All authors contributed to the article and approved the submitted version.

## Conflict of Interest

The authors declare that the research was conducted in the absence of any commercial or financial relationships that could be construed as a potential conflict of interest.

## Publisher's Note

All claims expressed in this article are solely those of the authors and do not necessarily represent those of their affiliated organizations, or those of the publisher, the editors and the reviewers. Any product that may be evaluated in this article, or claim that may be made by its manufacturer, is not guaranteed or endorsed by the publisher.

## Footnotes

^1^Ren, J., Zhou, F., Li, X., Ma, S., Jiang, Y., and Wu, C. (under revision). Robust Bayesian variable selection for gene-environment interactions. *Biometrics*.
