# Supplementary material for: Identifying Gene–Environment Interactions With Robust Marginal Bayesian Variable Selection
Source: Front Genet. 2021 Dec 8;12:667074. doi: 10.3389/fgene.2021.667074 (PMC8693717; doi:10.3389/fgene.2021.667074)
Supplement: Supplementary file 1 [file Presentation_1.pdf]

Supplementary Material for "Identifying Gene–Environment Interactions With Robust Marginal Bayesian Variable Selection" by Xi Lu, Kun Fan, Jie Ren and Cen Wu

A Additional simulation results

A.1 Identification results in simulation

Table 4: Simulation results of the second setting. AUC (mean of AUC), SD (sd of AUC) based on 100 replicates. n=200, p=500, q=4 and $m = 3$.

		BL	BLSS	LADBL	LADBLSS
Error 1	AUC	0.9089	0.9881	0.9148	0.9888
N(0,1)	SD	0.0059	0.0019	0.0051	0.0037
Error 2	AUC	0.8187	0.9255	0.8877	0.9769
$t(2)$	SD	0.0142	0.0524	0.0057	0.0048
Error 3	AUC	0.5333	0.5533	0.8239	0.9459
Lognormal(0,2)	SD	0.0096	0.0656	0.1045	0.0162
Error 4	AUC	0.8113	0.9122	0.9111	0.9849
90%N(0,1)+10%Cauchy(0,1)	SD	0.0166	0.0502	0.0083	0.0033
Error 5	AUC	0.7425	0.8086	0.9076	0.9856
80%N(0,1)+20%Cauchy(0,1)	SD	0.0241	0.0746	0.0065	0.0024

Table 5: Identification results of the second setting with Top100 method. mean(sd) based on 100 replicates. n=200, p=500, q=4 and $m = 3$.

		Main	Interaction	Total
Error 1	BL	7.50(0.86)	6.70(1.49)	14.20(1.83)
N(0,1)	BLSS	7.60(0.67)	10.20(0.09)	17.80(1.32)
	LADBL	7.67(0.66)	6.83(1.82)	14.5(1.96)
	LADBLSS	7.63(0.56)	9.97(1.54)	17.6(1.67)
Error 2	BL	5.83(2.21)	3.47(1.57)	9.30(2.98)
$t(2)$	BLSS	6.33(2.09)	7.57(3.15)	13.90(4.73)
	LADBL	7.07(0.94)	5.97(1.61)	13.03(1.96)
	LADBLSS	7.40(0.62)	9.20(1.94)	16.60(2.11)
Error 3	BL	0.77(0.86)	0.73(0.94)	1.50(1.11)
Lognormal(0,2)	BLSS	0.57(1.01)	0.67(1.06)	1.23(1.77)
	LADBL	5.90(1.65)	3.50(1.96)	9.40(2.43)
	LADBLSS	5.67(1.73)	9.00(2.35)	14.67(3.73)
Error 4	BL	6.03(2.19)	4.40(2.44)	10.43(4.17)
90%N(0,1)	BLSS	6.03(2.57)	8.00(3.33)	14.03(5.76)
+10%Cauchy(0,1)	LADBL	7.27(0.91)	6.87(1.48)	14.13(1.74)
	LADBLSS	7.53(0.63)	10.00(1.43)	17.53(1.57)
Error 5	BL	5.53(2.45)	3.63(2.19)	9.16(4.13)
80%N(0,1)	BLSS	5.07(2.57)	6.73(3.37)	11.80(5.65)
+20%Cauchy(0,1)	LADBL	7.47(0.97)	5.43(1.77)	12.90(2.04)
	LADBLSS	7.37(0.85)	10.47(1.46)	17.83(1.91)

Table 6: Simulation results of the third setting. AUC (mean of AUC), SD (sd of AUC) based on 100 replicates. n=200, p=500, q=4 and $m = 3$.

		BL	BLSS	LADBL	LADBLSS
Error 1	AUC	0.9158	0.9895	0.9251	0.9878
N(0,1)	SD	0.0041	0.0022	0.0054	0.0028
Error 2	AUC	0.8323	0.9461	0.8972	0.9833
$t(2)$	SD	0.0117	0.0342	0.0062	0.0028
Error 3	AUC	0.5268	0.5531	0.8415	0.9595
Lognormal(0,2)	SD	0.0127	0.0590	0.0107	0.0156
Error 4	AUC	0.8261	0.9323	0.9245	0.9889
90%N(0,1)+10%Cauchy(0,1)	SD	0.0191	0.0352	0.0056	0.0034
Error 5	AUC	0.7533	0.8591	0.9204	0.9862
80%N(0,1)+20%Cauchy(0,1)	SD	0.0201	0.0657	0.0067	0.0114

Table 7: Identification results of the third setting with Top100 method. mean(sd) based on 100 replicates. n=200, p=500, q=4 and $m = 3$.

		Main	Interaction	Total
Error 1	BL	7.70(0.47)	6.80(1.63)	14.50(1.79)
N(0,1)	BLSS	7.63(0.72)	10.93(0.98)	18.57(1.22)
	LADBL	7.70(0.75)	7.33(1.95)	15.03(2.14)
	LADBLSS	7.87(0.35)	10.33(1.35)	18.20(1.45)
Error 2	BL	6.57(1.87)	4.47(1.69)	11.03(2.88)
$t(2)$	BLSS	6.60(1.57)	8.40(2.51)	15.00(3.68)
	LADBL	7.57(0.62)	5.77(1.50)	13.33(1.77)
	LADBLSS	7.43(0.68)	9.30(2.15)	16.73(2.43)
Error 3	BL	0.50(0.73)	0.83(1.02)	1.33(1.47)
Lognormal(0,2)	BLSS	0.70(0.99)	0.40(0.86)	1.10(1.54)
	LADBL	6.13(2.05)	3.80(1.39)	9.93(1.32)
	LADBLSS	6.63(1.16)	10.10(1.73)	16.73(2.52)
Error 4	BL	5.73(2.82)	4.30(2.64)	10.03(5.11)
90%N(0,1)	BLSS	5.73(3.02)	7.67(4.19)	13.40(7.05)
+10%Cauchy(0,1)	LADBL	7.80(0.48)	6.87(1.61)	14.67(1.54)
	LADBLSS	7.83(0.38)	10.50(1.25)	18.33(1.39)
Error 5	BL	5.60(2.61)	2.93(2.23)	8.53(4.27)
80%N(0,1)	BLSS	5.27(2.27)	6.90(3.64)	12.17(5.66)
+20%Cauchy(0,1)	LADBL	7.87(0.35)	6.87(1.45)	14.73(1.46)
	LADBLSS	7.70(0.53)	10.70(1.12)	18.40(1.28)

B Estimation results for data analysis

Table 8: Analysis of the NHS T2D data using LADBLSS.

			Interactions				
SNP	Gene*	Main Effects	age	act	trans	ceraf	chol
rs17011106	WDFY4	-0.024					
rs7077294	KIAA1217						-0.0491
rs7093682	RP11-170M17.1				-0.1239		
rs17011106	WDFY4	-0.0953					
rs10826028	MIR3924					-0.0524	
rs4748996	THNSL1						0.0064
rs2646392	KRT8P37	0.0148					
rs7904629	RP11-170M17.1				-0.0592		
rs1244416	ATP5C1						0.0851
rs4838643	WDFY4	-0.0051					
rs1916458	RP11-170M17.1				-0.0264		
rs1537615	RP11-526P5.2	0.0477					
rs2765398	KRT8P37	-0.0157					
rs4317891	CELF2		0.0647				
rs7922793	LINC00845	-0.0345					
rs1916412	RP11-170M17.1				-0.0614		
rs1916411	RP11-170M17.1				-0.0448		
rs4747800	KRT8P37	0.0036					
rs11258040	CAMK1D					-0.0983	
rs1984275	RP11-319F12.2					0.0065	
rs17432763	MIR5100						-0.0677
rs10796113	FRMD4A	-0.0931					
rs224765	RP11-490O24.2		-0.0521				
rs6482387	KIAA1217						0.011
rs1492608	ENKUR						-0.0287
rs11257323	ECHDC3						0.0084
rs4434904	KIAA1217						-0.0374
rs10994364	ANK3		0.1086				
rs12220246	KIAA1462				0.0371		
rs11010390	RP11-309N24.1		0.0271				
rs10828584	KIAA1217	0.087					
rs10857590	ARHGAP22				-0.1379		
rs1537616	RP11-526P5.2	0.086					
rs17295031	KIAA1462				-0.0468		
rs10905778	RP11-271F18.4						0.0055
rs7093161	SNRPEP8		-0.0577				0.0107
rs2377872	CHAT					-0.0213	

Continued on the next page

Table 8: Continued from the previous page.

SNP	Gene*	Main Effects	Interactions				
			age	act	trans	ceraf	chol
rs1916409	RP11-170M17.1				-0.0642		
rs2245456	MALRD1		-0.0042				
rs787116	RP11-478H13.1		0.0259				
rs2817825	RP11-492M23.2		0.0278				
rs11255338	KIN						-0.0401
rs17011115	WDFY4	-0.0045					
rs11010821	Y-RNA	-0.025					
rs2532760	RP11-492M23.2		0.0272				
rs10821773	ANK3		-0.0107				
rs17454012	CELF2		-0.1076				
rs4372368	RP11-478B11.2		-0.0449				
rs1916420	RP11-170M17.1				0.0885		
rs2446588	FRMD4A		-0.0142				
rs10995687	RP11-170M17.1				0.1065		
rs161279	RP11-192P3.5				0.0333		
rs161279	ZEB1				0.0333		
rs161258	ZEB1				0.0362		
rs10509149	TMEM26						-0.0428
rs3740000	LINC00837	-0.106					
rs17314489	ZNF365		0.1543				
rs17453876	CELF2		0.0518				
rs10793451	ZNF485				-0.1028		
rs4749527	KIAA1462				0.0093		
rs12570207	SEPHS1		-0.0329				
rs902904	THNSL1						-0.0885
rs7921813	CAMK1D		0.0063				
rs10218945	SNRPEP8						-0.0351
rs2804551	RP11-492M23.2	0.0616					
rs12266433	CELF2		-0.0301				
rs16919385	PLXDC2						0.0112
rs4750039	CELF2		0.0333				
rs12249964	KIAA1217						0.0607
rs4745829	RP11-170M17.1				-0.0252		
rs11257932	CAMK1D		0.0256				
rs10827602	RP11-810B23.1			0.0167			
rs7081466	RP11-526P5.2					0.0267	
rs12256642	THNSL1						-0.0258
rs2796304	RP11-492M23.2		0.0875				
rs10826964	ZEB1				0.0316		
rs11257933	CAMK1D		0.0547				

Continued on the next page

Table 8: Continued from the previous page.

SNP	Gene*	Main Effects	Interactions				
			age	act	trans	ceraf	chol
rs17432532	MIR5100						0.017
rs10826899	UBE2V2P1					-0.0234	
rs11592473	UBE2V2P1					-0.0642	
rs12764778	OR13A1		0.0263				
rs12762462	GPR158				-0.0153		
rs1011763	MIR3924					0.1871	
rs1916450	RP11-170M17.1				-0.1767		
rs1917814	CHAT					-0.0194	
rs6602809	DCLRE1CP1	0.0043					
rs923757	THNSL1						0.0226
rs7092368	RP11-526P5.2					-0.0531	
rs6602806	DCLRE1CP1	0.0527					
rs6602806	ACBD7	0.0527					
rs10994308	ANK3						-0.0124
rs224699	RP11-490O24.2		-0.0351				
rs7083349	KIAA1217	-0.0651					
rs10828905	RNU6-632P		-0.0799				
rs10764441	KIAA1217	-0.0447					
rs10752217	CELF2		0.02				
rs17566968	CDC123						-0.057
rs7093183	KIAA1217						-0.0422
rs2887230	RP11-478H13.3	0.0864					
rs1761379	ZEB1				0.1187		
rs7097429	ALOX5		0.0789				

∗ Genes that SNPs belong to or are the closest to.

Table 9: Inclusion probability of the NHS T2D data using LADBLSS.

SNP	Gene*	Main Effects	age	act	trans	ceraf	chol
rs17011106	WDFY4	0.9930					
rs7077294	KIAA1217						0.9736
rs7093682	RP11-170M17.1				0.9938		
rs17011106	WDFY4	0.9900					
rs10826028	MIR3924					0.9612	
rs4748996	THNSL1						0.9834
rs2646392	KRT8P37	0.9818					
rs7904629	RP11-170M17.1				0.9646		
rs1244416	ATP5C1						0.9656
rs4838643	WDFY4	0.9768					

Continued on the next page

Table 9: Continued from the previous page.

SNP	Gene*	Main Effects	age	act	trans	ceraf	chol
rs1916458	RP11-170M17.1				0.9832		
rs1537615	RP11-526P5.2	0.9956					
rs2765398	KRT8P37	0.9756					
rs4317891	CELF2		1.000				
rs7922793	LINC00845	0.9744					
rs1916412	RP11-170M17.1				0.9774		
rs1916411	RP11-170M17.1				0.9700		
rs4747800	KRT8P37	0.9840					
rs11258040	CAMK1D					0.9738	
rs1984275	RP11-319F12.2					0.9952	
rs17432763	MIR5100						0.9862
rs10796113	FRMD4A	0.9636					
rs224765	RP11-490O24.2		0.9710				
rs6482387	KIAA1217						0.9638
rs1492608	ENKUR						0.9680
rs11257323	ECHDC3						0.9892
rs4434904	KIAA1217						0.9716
rs10994364	ANK3		0.9942				
rs12220246	KIAA1462				0.9610		
rs11010390	RP11-309N24.1		0.9820				
rs10828584	KIAA1217	0.9752					
rs10857590	ARHGAP22				0.9848		
rs1537616	RP11-526P5.2	0.9944					
rs17295031	KIAA1462				0.9816		
rs10905778	RP11-271F18.4						0.9988
rs7093161	SNRPEP8		0.9542				0.9902
rs2377872	CHAT					0.9728	
rs1916409	RP11-170M17.1				0.9612		
rs2245456	MALRD1		0.9630				
rs787116	RP11-478H13.1		0.9638				
rs2817825	RP11-492M23.2		0.9550				
rs11255338	KIN						0.9964
rs17011115	WDFY4	0.9712					
rs11010821	Y-RNA	0.9916					
rs2532760	RP11-492M23.2		0.9720				
rs10821773	ANK3		0.9586				
rs17454012	CELF2		0.9998				
rs4372368	RP11-478B11.2		0.9618				
rs1916420	RP11-170M17.1				0.9672		
rs2446588	FRMD4A		0.9724				
rs10995687	RP11-170M17.1				0.9588		

Continued on the next page

Table 9: Continued from the previous page.

SNP	Gene*	Main Effects	age	act	trans	ceraf	chol
rs161279	RP11-192P3.5				0.9770		
rs161279	ZEB1				0.9770		
rs161258	ZEB1				0.9876		
rs10509149	TMEM26						0.9726
rs3740000	LINC00837	0.9964					
rs17314489	ZNF365		0.9866				
rs17453876	CELF2		0.9952				
rs10793451	ZNF485				0.9604		
rs4749527	KIAA1462				0.9794		
rs12570207	SEPHS1		0.9698				
rs902904	THNSL1						0.9884
rs7921813	CAMK1D		0.9998				
rs10218945	SNRPEP8						0.9612
rs2804551	RP11-492M23.2	0.9848					
rs12266433	CELF2		0.9618				
rs16919385	PLXDC2						0.9806
rs4750039	CELF2		0.9910				
rs12249964	KIAA1217						0.9558
rs4745829	RP11-170M17.1				0.9940		
rs11257932	CAMK1D		0.9826				
rs10827602	RP11-810B23.1			0.9728			
rs7081466	RP11-526P5.2					0.9714	
rs12256642	THNSL1						0.9616
rs2796304	RP11-492M23.2		0.9928				
rs10826964	ZEB1				0.9592		
rs11257933	CAMK1D		0.9726				
rs17432532	MIR5100						0.9834
rs10826899	UBE2V2P1					0.9784	
rs11592473	UBE2V2P1					0.9864	
rs12764778	OR13A1		0.9894				
rs12762462	GPR158				0.9636		
rs1011763	MIR3924					0.9954	
rs1916450	RP11-170M17.1				0.9820		
rs1917814	CHAT					0.9670	
rs6602809	DCLRE1CP1	0.9614					
rs923757	THNSL1						0.9964
rs7092368	RP11-526P5.2					0.9868	
rs6602806	DCLRE1CP1	0.9912					
rs6602806	ACBD7	0.9912					
rs10994308	ANK3						0.9542
rs224699	RP11-490O24.2		0.9768				

Continued on the next page

Table 9: Continued from the previous page.

SNP	Gene*	Main Effects	age	act	trans	ceraf	chol
rs7083349	KIAA1217	0.9986					
rs10828905	RNU6-632P		0.9626				
rs10764441	KIAA1217	0.9896					
rs10752217	CELF2		0.9762				
rs17566968	CDC123						0.9838
rs7093183	KIAA1217						0.9954
rs2887230	RP11-478H13.3	0.9588					
rs1761379	ZEB1				0.9682		
rs7097429	ALOX5		0.9974				

∗ Genes that SNPs belong to or are the closest to.

C Posterior inference

C.1 LADBL

C.1.1 Hierarchical model specification

$$
\begin{aligned}
Y_i = E_i\alpha + C_i\gamma + X_{ij}\beta_j + \tilde{W}_i\eta_j + \tau^{-1/2}\xi_2\sqrt{v_i}z_i \quad i = 1, \ldots, n \\
v_i|\tau \overset{iid}{\sim} \tau\text{exp}(-\tau v_i) \quad i = 1, \ldots, n \\
z_i \overset{iid}{\sim} \text{N}(0,1) \quad i = 1, \ldots, n \\
\beta_j|s_1 \sim \frac{1}{\sqrt{2\pi s_1}}\text{exp}(-\frac{\beta_j^2}{2s_1}) \\
s_1|\varphi_1^2 \sim \frac{\varphi_1^2}{2}\text{exp}(-\frac{\varphi_1^2}{2}s_1) \\
\eta_{jk}|s_{2k} \overset{iid}{\sim} \frac{1}{\sqrt{2\pi s_{2k}}}\text{exp}(-\frac{\eta_{jk}^2}{2s_{2k}}) \quad k = 1, \ldots, q \\
s_{2k}|\varphi_2^2 \overset{iid}{\sim} \frac{\varphi_2^2}{2}\text{exp}(-\frac{\varphi_2^2}{2}s_{2k}) \quad k = 1, \ldots, q \\
\alpha_k \overset{iid}{\sim} \frac{1}{\sqrt{(2\pi\alpha_0)}}\text{exp}(-\frac{\alpha_k^2}{2\alpha_0}) \quad k = 1, \ldots, q \\
\gamma_t \overset{iid}{\sim} \frac{1}{\sqrt{(2\pi\gamma_0)}}\text{exp}(-\frac{\gamma_t^2}{2\gamma_0}) \quad t = 1, \ldots, m \\
\tau \sim \text{Gamma}(a, b) \\
\varphi_1^2 \sim \text{Gamma}(c_1, d_1) \\
\varphi_2^2 \sim \text{Gamma}(c_2, d_2)
\end{aligned}
$$

C.1.2 Gibbs Sampler

Let $\mu_{(-\alpha_k)} = E(y_i) - E_{ik}\alpha_k$, then

$$
\begin{aligned}
&\pi(\alpha_k|\text{rest}) \\
&\quad \propto \pi(Y|\cdot)\pi(\alpha_k) \\
&\quad \propto \exp\Big\{-\sum_{i=1}^{n}\frac{(y_i - E_i\alpha - C_i\gamma - X_{ij}\beta_j - \tilde{W}_i\eta_j)^2}{2\tau^{-1}\xi_2^2 v_i}\Big\} \times \exp(-\frac{\alpha_k^2}{2\alpha_0}) \\
&\quad \propto \exp\Big\{-\frac{1}{2}\big[(\sum_{i=1}^{n}\frac{\tau E_{ik}^2}{\xi_2^2 v_i} + \frac{1}{\alpha_0})\alpha_k^2 - 2\sum_{i=1}^{n}\frac{\tau(y_i - \mu_{(-\alpha_k)})E_{ik}}{\xi_2^2 v_i}\alpha_k\big]\Big\}.
\end{aligned}
$$

Hence, $\alpha_k|\text{rest}\sim N(\mu_{\alpha_k}, \sigma^2_{\alpha_k})$, where

$$\mu_{\alpha_k} = (\sum_{i=1}^{n} \frac{\tau(y_i - \mu_{(-\alpha_k)})E_{ik}}{\xi_2^2 v_i})\sigma^2_{\alpha_k},$$
$$\sigma^2_{\alpha_k} = (\sum_{i=1}^{n} \frac{\tau E_{ik}^2}{\xi_2^2 v_i} + \frac{1}{\alpha_0})^{-1}.$$

Let $\mu_{(-\gamma_t)} = E(y_i) - C_{it}\gamma_t$, So $\gamma_t|\text{rest}\sim N(\mu_{\gamma_k}, \sigma^2_{\gamma_t})$, where

$$\mu_{\gamma_t} = (\sum_{i=1}^{n} \frac{\tau(y_i - \mu_{(-\gamma_t)})C_{it}}{\xi_2^2 v_i})\sigma^2_{\gamma_t},$$
$$\sigma^2_{\gamma_t} = (\sum_{i=1}^{n} \frac{\tau C_{it}^2}{\xi_2^2 v_i} + \frac{1}{\gamma_0})^{-1}.$$

Let $\mu_{(-\beta_j)} = E(y_i) - X_{ij}\beta_j$, then

$$\begin{aligned}
&\pi(\beta_j|\text{rest}) \\
&\quad\propto \pi(y|\cdot)\pi(\beta_j|s_1) \\
&\quad\propto \exp\Big\{ - \sum_{i=1}^{n} \frac{(y_i - E_i\alpha - C_i\gamma - X_{ij}\beta_j - \tilde{W}_i\eta_j)^2}{2\tau^{-1}\xi_2^2 v_i}\Big\} \times \exp(-\frac{\beta_j^2}{2s_1}) \\
&\quad\propto \exp\Big\{ - \frac{1}{2}\big[(\sum_{i=1}^{n} \frac{\tau X_{ij}^2}{\xi_2^2 v_i} + \frac{1}{s_1})\beta_j^2 - 2\sum_{i=1}^{n} \frac{\tau(y_i - \mu_{(-\beta_j)})X_{ij}}{\xi_2^2 v_i}\beta_j\big]\Big\}.
\end{aligned}$$

So, $\beta_j|\text{rest}\sim N(\mu_{\beta_j}, \sigma^2_{\beta_j})$ with

$$\mu_{\beta_j} = (\sum_{i=1}^{n} \frac{\tau(y_i - \mu_{(-\beta_j)})X_{ij}}{\xi_2^2 v_i})\sigma^2_{\beta_j},$$
$$\sigma^2_{\beta_j} = (\sum_{i=1}^{n} \frac{\tau X_{ij}^2}{\xi_2^2 v_i} + \frac{1}{s_1})^{-1}.$$

Let $\mu_{(-\eta_{jk})} = E(y_i) - W_{ik}\eta_{jk}$, then $\eta_{jk}|\text{rest}\sim N(\mu_{\eta_{jk}}, \sigma^2_{\eta_{jk}})$, where

$$\mu_{\eta_{jk}} = (\sum_{i=1}^{n} \frac{\tau(y_i - \mu_{(-\eta_{jk})})\tilde{W}_{ik}}{\xi_2^2 v_i})\sigma^2_{\eta_{jk}},$$
$$\sigma^2_{\beta_j} = (\sum_{i=1}^{n} \frac{\tau \tilde{W}_{ik}^2}{\xi_2^2 v_i} + \frac{1}{s_{2k}})^{-1}.$$

The full conditional posterior distribution of s_1 is:

$$
\begin{aligned}
&s_1|\text{rest}\\
&\quad \propto \pi(\beta_j|s_1)\pi(s_1|\varphi_1^2)\\
&\quad \propto \frac{1}{\sqrt{s_1}}\exp(-\frac{\varphi_1^2}{2}s_1)\exp(-\frac{\beta_j^2}{2s_1})\\
&\quad \propto \frac{1}{\sqrt{s_1}}\exp\Big\{-\frac{1}{2}[\varphi_1^2 s_1+\frac{\beta_j^2}{s_1}]\}.
\end{aligned}
$$

Therefore, $s_1^{-1}|\text{rest}\sim$ Inverse-Gaussian$(\sqrt{\frac{\varphi_1^2}{\beta_j^2}},\varphi_1^2)$.

Similiarly, for $s_{2k}(k=1,\dots,q)$, the posterior distribution for is $s_{2k}^{-1}|\text{rest}\sim$ Inverse-Gaussian$(\sqrt{\frac{\varphi_2^2}{\eta_{jk}^2}},\varphi_2^2)$.

The full conditional posterior distribution of φ_1^2 is:

$$
\begin{aligned}
&\varphi_1^2|\text{rest}\\
&\quad \propto \pi(s_1|\varphi_1^2)\pi(\varphi_1^2)\\
&\quad \propto \frac{\varphi_1^2}{2}\exp(-\frac{\varphi_1^2 s_1}{2})(\varphi_1^2)^{c_1-1}\exp(-d_1\varphi_1^2)\\
&\quad \propto (\varphi_1^2)^{c_1}\exp\Big(-\varphi_1^2(s_1/2+d_1)\Big).
\end{aligned}
$$

Therefore, the posterior distribution for φ_1^2 is Gamma$(c_1+1, s_1/2+d_1)$.

The full conditional posterior distribution of φ_2^2 is:

$$
\begin{aligned}
&\varphi_2^2|\text{rest}\\
&\quad \propto \pi(s_2|\varphi_2^2)\pi(\varphi_2^2)\\
&\quad \propto \prod_{k=1}^{q}\frac{\varphi_2^2}{2}\exp(-\frac{\varphi_2^2 s_{2k}}{2})(\varphi_2^2)^{c_2-1}\exp(-d_2\varphi_2^2)\\
&\quad \propto (\varphi_2^2)^{q+c_2-1}\exp\Big(-\varphi_2^2(\sum_{k=1}^{q}\frac{s_{2k}}{2}+d_2)\Big).
\end{aligned}
$$

The posterior distribution for φ_2^2 is Gamma$(c_2+q, \sum_{k=1}^{q} s_{2k}/2+d_2)$.

The full conditional posterior distribution of τ:

$$
\begin{aligned}
&\tau|\text{rest}\\
&\quad \propto \pi(v|\tau)\pi(\tau)\pi(Y|\cdot)\\
&\quad \propto \tau^{n/2}\exp\Big\{-\sum_{i=1}^{n}\frac{(y_i-E_i\alpha-C_i\gamma-X_{ij}\beta_j-\tilde{W}_i\eta_j)^2}{2\tau^{-1}\xi_2^2 v_i}\Big\}\\
&\quad \times \tau^n\exp(-\tau\sum_{i=1}^{n}v_i)\tau^{a-1}\exp(-b\tau)\\
&\quad \propto \tau^{a+\frac{3}{2}n-1}\exp\Big\{-\tau\big[\sum_{i=1}^{n}(\frac{(y_i-E_i\alpha-C_i\gamma-X_{ij}\beta_j-\tilde{W}_i\eta_j)^2}{2\xi_2^2 v_i}+v_i)+b\big]\Big\}.
\end{aligned}
$$

Therefore, $\tau|\text{rest}\sim\text{Gamma}(a+\frac{3}{2}n,\ \big[\sum_{i=1}^{n}(\frac{(y_i-E_i\alpha-C_i\gamma-X_{ij}\beta_j-\tilde{W}_i\eta_j)^2}{2\xi_2^2 v_i}+v_i)+b\big])$.

The full conditional posterior distribution of v_i is:

$$\begin{aligned}
&v_i|\text{rest}\\
&\quad\propto \pi(v|\tau)\pi(y|\cdot)\\
&\quad\propto \frac{1}{\sqrt{v_i}}\exp\Big\{-\frac{(y_i-E_i\alpha-C_i\gamma-X_{ij}\beta_j-\tilde{W}_i\eta_j)^2}{2\tau^{-1}\xi_2^2 v_i}\Big\}\times\exp(-\tau v_i)\\
&\quad\propto \frac{1}{\sqrt{v_i}}\exp\Big\{-\frac{1}{2}\big[(2\tau)v_i+\frac{\tau(y_i-E_i\alpha-C_i\gamma-X_{ij}\beta_j-\tilde{W}_i\eta_j)^2}{\xi_2^2 v_i}\big]\Big\}.
\end{aligned}$$

Therefore,

$$\frac{1}{v_i}|\text{rest}\sim\ \text{Inverse-Gaussian}(\sqrt{\frac{2\xi_2^2}{(y_i-E_i\alpha-C_i\gamma-X_{ij}\beta_j-\tilde{W}_i\eta_j)^2}},\ 2\tau).$$

C.2 BLSS

C.2.1 Hierarchical model specification

$$\begin{gathered}
Y\propto(\sigma^2)^{-\frac{n}{2}}\exp\left\{-\frac{1}{2\sigma^2}\sum_{i=1}^{n}(y_i-E_i\alpha-C_i\gamma-X_{ij}\beta_j-\tilde{W}_i\eta_j)^2\right\}\\
\alpha\sim\text{N}_q(0,\,\Sigma_{\alpha 0})\\
\gamma\sim\text{N}_m(0,\,\Sigma_{\gamma 0})\\
\beta_j|\pi_c,\tau_c^2,\sigma^2\sim(1-\pi_c)\,\text{N}\left(0,\,\sigma^2\tau_c^2\right)+\pi_c\,\delta_0(\beta_j)\quad j=1,\ldots,p\\
\eta_{jk}|\pi_e,\tau_{ek}^2,\sigma^2\overset{iid}{\sim}(1-\pi_e)\,\text{N}\left(0,\,\sigma^2\tau_{ek}^2\right)+\pi_e\,\delta_0(\eta_{jk})\quad j=1,\ldots,p,\,k=1,\ldots,q\\
\tau_c^2|\lambda_c^2\sim\text{Gamma}(1,\,\frac{\lambda_c^2}{2})\\
\tau_{ek}^2|\lambda_e^2\overset{iid}{\sim}\text{Gamma}(1,\,\frac{\lambda_e^2}{2})\quad k=1,\ldots,q\\
\pi_c\sim\text{Beta}\,(r_c,\,u_c)\\
\pi_e\sim\text{Beta}\,(r_e,\,u_e)\\
\lambda_c^2\sim\text{Gamma}\,(a_c,\,b_c)\\
\lambda_e^2\sim\text{Gamma}\,(a_e,\,b_e)\\
\sigma^2\sim\text{Inverse-Gamma}\,(s,\,h)
\end{gathered}$$

C.2.2 Gibbs Sampler

Denote $\mu_{(-\alpha)}=\text{E}(Y)-E\alpha$, then $\alpha|\text{rest}\sim\text{N}(\mu_\alpha,\,\Sigma_\alpha)$, where

$$\begin{aligned}
\mu_\alpha&=\Sigma_\alpha(\frac{1}{\sigma^2}(Y-\mu_{(-\alpha)})^\top E)^\top,\\
\Sigma_\alpha&=\left(\frac{1}{\sigma^2}E^\top E+\Sigma_{\alpha 0}^{-1}\right)^{-1}.
\end{aligned}$$

Denote $\mu_{(-\gamma)} = \mathrm{E}(Y) - C\gamma$, then $\gamma|\text{rest} \sim \mathrm{N}(\mu_\gamma,\, \Sigma_\gamma)$, where

$$\mu_\gamma = \Sigma_\gamma(\frac{1}{\sigma^2}(Y - \mu_{(-\gamma)})^\top C)^\top,$$
$$\Sigma_\gamma = \left(\frac{1}{\sigma^2}C^\top C + \Sigma_{\gamma 0}^{-1}\right)^{-1}.$$

Denote $\mu_{(-\beta_j)} = \mathrm{E}(Y) - X_j\beta_j$, then $\beta_j|\text{rest} \sim (1-l_c)\mathrm{N}(\mu_{\beta_j},\, \sigma^2\Sigma_{\beta_j}) + l_c\delta_0(\beta_j)$, where

$$\mu_{\beta_j} = \Sigma_{\beta_j}X_j^\top(Y - \mu_{(-\beta_j)}),$$
$$\Sigma_{\beta_j} = \left(X_j^\top X_j + \frac{1}{\tau_c^2}\right)^{-1},$$
$$l_c = \frac{\pi_c}{\pi_c + (1-\pi_c)(\tau_c^2)^{-1/2}|\Sigma_{\beta_j}|^{1/2}\exp\{\frac{1}{2\sigma^2}\Sigma_{\beta_j}||X_j^\top(Y - \mu_{(-\beta_j)})||_2^2\}}.$$

Denote $\mu_{(-\eta_{jk})} = \mathrm{E}(Y) - \tilde{W}_k\eta_{jk}$, then $\eta_{jk}|\text{rest} \sim (1-l_{ek})\mathrm{N}(\mu_{\eta_{jk}},\, \sigma^2\Sigma_{\eta_{jk}}) + l_e\delta_0(\eta_{jk})$, where

$$\mu_{\eta_{jk}} = \Sigma_{\eta_{jk}}\tilde{W}_k^\top(Y - \mu_{(-\eta_{jk})}),$$
$$\Sigma_{\eta_{jk}} = \left(\tilde{W}_k^\top\tilde{W}_k + \frac{1}{\tau_{ek}^2}\right)^{-1},$$
$$l_e = \frac{\pi_e}{\pi_e + (1-\pi_e)(\tau_{ek}^2)^{-1/2}|\Sigma_{\eta_{jk}}|^{1/2}\exp\{\frac{1}{2\sigma^2}\Sigma_{\eta_{jk}}||\tilde{W}_k^\top(Y - \mu_{(-\eta_{jk})})||_2^2\}}.$$

The posterior of τ_c^2 is:

$$\frac{1}{\tau_c^2}|\text{rest} \sim \begin{cases}\text{Inverse-Gamma}(1, \frac{\lambda_c^2}{2}) & \text{if } \beta_j = 0\\ \text{Inverse-Gaussian}(\sqrt{\frac{\sigma^2}{\beta_j^2}\lambda_c^2},\, \lambda_c^2) & \text{if } \beta_j \neq 0\end{cases}.$$

The posterior of τ_{ek}^2 is:

$$\frac{1}{\tau_{ek}^2}|\text{rest} \sim \begin{cases}\text{Inverse-Gamma}(1, \frac{\lambda_e^2}{2}) & \text{if } \eta_{jk} = 0\\ \text{Inverse-Gaussian}(\sqrt{\frac{\sigma^2}{\eta_{jk}^2}\lambda_e^2},\, \lambda_e^2) & \text{if } \eta_{jk} \neq 0\end{cases}.$$

λ_c^2 and λ_e^2 have Gamma posterior distributions:

$$\lambda_c^2|\text{rest} \sim \text{Gamma}(a_c + 1,\, \frac{\tau_c^2}{2} + b_c),$$
$$\lambda_e^2|\text{rest} \sim \text{Gamma}(a_e + q,\, \sum_{k=1}^q \frac{\tau_{ek}^2}{2} + b_e).$$

π_c and π_e have Gamma posterior distributions:

$$\pi_c|\text{rest} \sim \text{Beta}(r_c - \mathbf{I}_{\{\beta_j \neq 0\}} + 1,\ u_c + \mathbf{I}_{\{\beta_j \neq 0\}}),$$
$$\pi_e|\text{rest} \sim \text{Beta}(r_e - \sum_{k=1}^q \mathbf{I}_{\{\eta_{jk} \neq 0\}} + q,\ u_e + \sum_{k=1}^q \mathbf{I}_{\{\eta_{jk} \neq 0\}}).$$

$\sigma^2 \sim$ Inverse-Gamma$(\mu_{\sigma^2},\ \Sigma_{\sigma^2})$, where

$$\mu_{\sigma^2} = s + \frac{n + \mathbf{I}_{\{\beta_j \neq 0\}} + \sum_{k=1}^{q} \mathbf{I}_{\{\eta_{jk} \neq 0\}}}{2},$$
$$\Sigma_{\sigma^2} = h + \frac{(Y-\mu)^\top (Y-\mu) + (\tau_c^2)^{-1}\beta_j^2 + \sum_{k=1}^{q} (\tau_{ek}^2)^{-1} \eta_j^\top \eta_j}{2}.$$

C.3 BL

C.3.1 Hierarchical model specification

$$Y \propto (\sigma^2)^{-\frac{n}{2}} \exp\left\{ -\frac{1}{2\sigma^2} \sum_{i=1}^{n} (y_i - E_i\alpha - C_i\gamma - X_{ij}\beta_j - \tilde{W}_i \eta_j)^2 \right\}$$
$$\alpha \sim \mathrm{N}_q(0,\ \Sigma_{\alpha 0})$$
$$\gamma \sim \mathrm{N}_m(0,\ \Sigma_{\gamma 0})$$
$$\beta_j | \tau_c^2, \sigma^2 \sim \mathrm{N}\left(0,\ \sigma^2 \tau_c^2\right) \quad j = 1, \ldots, p$$
$$\eta_{jk} | \tau_{ek}^2, \sigma^2 \overset{iid}{\sim} \mathrm{N}\left(0,\ \sigma^2 \tau_{ek}^2\right) \quad j = 1, \ldots, p,\ k = 1, \ldots, q$$
$$\tau_c^2 | \lambda_c^2 \sim \exp(\frac{\lambda_c^2}{2})$$
$$\tau_{ek}^2 | \lambda_e^2 \overset{iid}{\sim} \exp(\frac{\lambda_e^2}{2}) \quad k = 1, \ldots, q$$
$$\lambda_c^2 \sim \mathrm{Gamma}\left(a_c,\ b_c\right)$$
$$\lambda_e^2 \sim \mathrm{Gamma}\left(a_e,\ b_e\right)$$
$$\sigma^2 \propto \frac{1}{\sigma^2}$$

C.3.2 Gibbs Sampler

Denote $\mu_{(-\alpha)} = \mathrm{E}(Y) - E\alpha$, then $\alpha|\text{rest} \sim \mathrm{N}(\mu_\alpha,\ \Sigma_\alpha)$, where

$$\mu_\alpha = \Sigma_\alpha (\frac{1}{\sigma^2} (Y - \mu_{(-\alpha)})^\top E)^\top,$$
$$\Sigma_\alpha = \left(\frac{1}{\sigma^2} E^\top E + \Sigma_{\alpha 0}^{-1} \right)^{-1}.$$

Denote $\mu_{(-\gamma)} = \mathrm{E}(Y) - C\gamma$, then $\gamma|\text{rest} \sim \mathrm{N}(\mu_\gamma,\ \Sigma_\gamma)$, where

$$\mu_\gamma = \Sigma_\gamma (\frac{1}{\sigma^2} (Y - \mu_{(-\gamma)})^\top C)^\top,$$
$$\Sigma_\gamma = \left(\frac{1}{\sigma^2} C^\top C + \Sigma_{\gamma 0}^{-1} \right)^{-1}.$$

Denote $\mu_{(-\beta_j)} = \mathrm{E}(Y) - X_j\beta_j$, then $\beta_j|\text{rest} \sim \mathrm{N}(\mu_{\beta_j},\, \sigma^2\Sigma_{\beta_j})$, where

$$\begin{aligned}\mu_{\beta_j} &= \Sigma_{\beta_j} X_j^\top (Y - \mu_{(-\beta_j)}),\\ \Sigma_{\beta_j} &= \left(X_j^\top X_j + \frac{1}{\tau_c^2}\right)^{-1}.\end{aligned}$$

Denote $\mu_{(-\eta_{jk})} = \mathrm{E}(Y) - \tilde{W}_k\eta_{jk}$, then $\eta_{jk}|\text{rest} \sim \mathrm{N}(\mu_{\eta_{jk}},\, \sigma^2\Sigma_{\eta_{jk}})$, where

$$\begin{aligned}\mu_{\eta_{jk}} &= \Sigma_{\eta_{jk}} \tilde{W}_k^\top (Y - \mu_{(-\eta_{jk})}),\\ \Sigma_{\eta_{jk}} &= \left(\tilde{W}_k^\top \tilde{W}_k + \frac{1}{\tau_{ek}^2}\right)^{-1}.\end{aligned}$$

The posterior of τ_c^2 is:

$$\frac{1}{\tau_c^2}|\text{rest} \sim \text{Inverse-Gaussian}(\sqrt{\frac{\sigma^2}{\beta_j^2}\lambda_c^2}, \lambda_c^2).$$

The posterior of τ_{ek}^2 is:

$$\frac{1}{\tau_{ek}^2}|\text{rest} \sim \text{Inverse-Gaussian}(\sqrt{\frac{\sigma^2}{\eta_{jk}^2}\lambda_e^2}, \lambda_e^2).$$

λ_c^2 and λ_e^2 have Gamma posterior distributions:

$$\begin{aligned}\lambda_c^2|\text{rest} &\sim \text{Gamma}(a_c + 1,\ \frac{\tau_c^2}{2} + b_c),\\ \lambda_e^2|\text{rest} &\sim \text{Gamma}(a_e + q,\ \sum_{k=1}^{q}\frac{\tau_{ek}^2}{2} + b_e).\end{aligned}$$

$\sigma^2 \sim \text{Inverse-Gamma}(\mu_{\sigma^2},\ \Sigma_{\sigma^2})$, where

$$\begin{aligned}\mu_{\sigma^2} &= \frac{n+1+q}{2},\\ \Sigma_{\sigma^2} &= \frac{(Y-\mu)^\top(Y-\mu) + (\tau_c^2)^{-1}\beta_j^2 + \sum_{k=1}^{q}(\tau_{ek}^2)^{-1}\eta_j^\top\eta_j}{2}.\end{aligned}$$
